# Musicianship-Related Structural and Functional Cortical Features Are Preserved in Elderly Musicians

**DOI:** 10.3389/fnagi.2022.807971

**Published:** 2022-03-25

**Authors:** Oana G. Rus-Oswald, Jan Benner, Julia Reinhardt, Céline Bürki, Markus Christiner, Elke Hofmann, Peter Schneider, Christoph Stippich, Reto W. Kressig, Maria Blatow

**Affiliations:** ^1^Department of Neuroradiology, Clinical Neuroscience Center, University Hospital Zurich, University of Zurich, Zürich, Switzerland; ^2^University Department of Geriatric Medicine FELIX PLATTER, Basel, Switzerland; ^3^Department of Neuroradiology, Heidelberg University Hospital, Heidelberg, Germany; ^4^Division of Diagnostic and Interventional Neuroradiology, Department of Radiology, University Hospital Basel, University of Basel, Basel, Switzerland; ^5^Department of Cardiology and Cardiovascular Research Institute Basel, University Hospital Basel, University of Basel, Basel, Switzerland; ^6^Department of Orthopedic Surgery and Traumatology, University Hospital of Basel, University of Basel, Basel, Switzerland; ^7^Centre for Systematic Musicology, University of Graz, Graz, Austria; ^8^Vitols Jazeps Latvian Academy of Music, Riga, Latvia; ^9^Academy of Music, University of Applied Sciences and Arts Northwestern Switzerland (FHNW), Basel, Switzerland; ^10^Department of Neuroradiology and Radiology, Kliniken Schmieder, Allensbach, Germany; ^11^Section of Neuroradiology, Department of Radiology and Nuclear Medicine, Neurocenter, Cantonal Hospital Lucerne, University of Lucerne, Lucerne, Switzerland

**Keywords:** musicians, musicianship, elderly, aging, auditory cortex, structural, functional, fMRI

## Abstract

**Background:**

Professional musicians are a model population for exploring basic auditory function, sensorimotor and multisensory integration, and training-induced neuroplasticity. The brain of musicians exhibits distinct structural and functional cortical features; however, little is known about how these features evolve during aging. This multiparametric study aimed to examine the functional and structural neural correlates of lifelong musical practice in elderly professional musicians.

**Methods:**

Sixteen young musicians, 16 elderly musicians (age >70), and 15 elderly non-musicians participated in the study. We assessed gray matter metrics at the whole-brain and region of interest (ROI) levels using high-resolution magnetic resonance imaging (MRI) with the Freesurfer automatic segmentation and reconstruction pipeline. We used BrainVoyager semiautomated segmentation to explore individual auditory cortex morphotypes. Furthermore, we evaluated functional blood oxygenation level-dependent (BOLD) activations in auditory and non-auditory regions by functional MRI (fMRI) with an attentive tone-listening task. Finally, we performed discriminant function analyses based on structural and functional ROIs.

**Results:**

A general reduction of gray matter metrics distinguished the elderly from the young subjects at the whole-brain level, corresponding to widespread natural brain atrophy. Age- and musicianship-dependent structural correlations revealed group-specific differences in several clusters including superior, middle, and inferior frontal as well as perirolandic areas. In addition, the elderly musicians exhibited increased gyrification of auditory cortex like the young musicians. During fMRI, the elderly non-musicians activated predominantly auditory regions, whereas the elderly musicians co-activated a much broader network of auditory association areas, primary and secondary motor areas, and prefrontal and parietal regions like, albeit weaker, the young musicians. Also, group-specific age- and musicianship-dependent functional correlations were observed in the frontal and parietal regions. Moreover, discriminant function analysis could separate groups with high accuracy based on a set of specific structural and functional, mainly temporal and occipital, ROIs.

**Conclusion:**

In conclusion, despite naturally occurring senescence, the elderly musicians maintained musicianship-specific structural and functional cortical features. The identified structural and functional brain regions, discriminating elderly musicians from non-musicians, might be of relevance for the aging musicians’ brain. To what extent lifelong musical activity may have a neuroprotective impact needs to be addressed further in larger longitudinal studies.

## Introduction

### The Musical Brain

Brain studies on professional musicians enabled effective investigations of basic auditory function, sensorimotor and multisensory integration, and training-induced neuroplasticity (Jäncke, 2009; Zatorre et al., 2012; Zatorre and Salimpoor, 2013; Zatorre, 2018). Structurally, musicians exhibit increased gray matter (GM) volume in motor, auditory, and visuospatial regions when compared to non-musicians (Gaser and Schlaug, 2003; James et al., 2014). In particular, the volume and gyrification of the auditory cortex (AC), primarily Heschl’s gyrus (HG), are known to be substantially increased in professional musicians (Schneider et al., 2002; Schneider et al., 2005b; Wengenroth et al., 2014). Additionally, as group comparison results might obscure relevant information on specific individual differences, mostly because of high inter-hemispheric and inter-individual variability (Schneider et al., 2002, 2005b; Golestani and Pallier, 2007; Seither-Preisler et al., 2014; Marie et al., 2015, 2016), attempts have been made to evaluate and classify individual AC size, shape, and gyrification patterns in musicians (Rademacher et al., 1993, 2001; Schneider et al., 2005b; Benner et al., 2017; Dalboni da Rocha et al., 2020). Hence, the extended size, shape, and individual diversity of AC structure in musicians have been associated to innate predispositions and, potentially, initial neuroplastic processes developed early in life (Seither-Preisler et al., 2014; Vaquero et al., 2016; Palomar-Garcia et al., 2020; for a review, see Altenmüller and Furuya (2016)).

Interestingly, cortical pattern variations in musicians are not confined to auditory areas or GM. Hence, Bangert and Schlaug (2006) found a likewise increased size and a more complex gyrification pattern of the motor hand region (homunculus “hand knob”) in professional pianists, while other studies reported that professional musicians showed enhanced information transmission efficiency in the white matter (WM) network connecting AC regions (Li et al., 2014; Elmer et al., 2016). Functionally, when exposed only to simple tones, corresponding auditory activations follow the extended shape of the individual AC in musicians (Da Costa et al., 2011). In addition to the AC, musicians, in contrast to non-musicians, co-activate a broad cortical network of frontal and parietal areas along the two main auditory processing pathways proposed by Rauschecker (2018), i.e., the dorsal and ventral processing streams, responsible for tonal context processing and tone identification, respectively (Baumann et al., 2007; Wengenroth et al., 2014).

### The Aging Brain

Aging induces a variety of structural and functional brain changes and is not restricted to certain regions or metrics (Gonzalez-Escamilla et al., 2018; Ebaid and Crewther, 2020). Hence, it can affect brain macrostructure in the form of cortical thinning or reduction in GM volume and WM integrity (Resnick et al., 2003; Salat et al., 2004; Hogstrom et al., 2013; Hafkemeijer et al., 2014; Ramanoel et al., 2018; Topiwala et al., 2019; Zheng et al., 2019). Functional disruptions during aging can imply specificity loss of brain areas within the visual or motor system, impaired recruitment of task-relevant brain regions, e.g., the medial temporal lobe (MTL), for memory-associated tasks, and disturbed network connectivity under rest conditions, i.e., impaired default mode network in the elderly (Cabeza, 2002; Gutchess et al., 2005; Brown et al., 2018; Chen et al., 2019; Jockwitz et al., 2019; Malagurski et al., 2020). Overall, brain changes linked to aging refer mostly to structural atrophy and functional degradation and connectivity disruption, and have been detected at both global and region-specific levels, e.g., the often-reported hippocampal atrophy (Schroeder et al., 2017). However, aging theories postulate that most probably because of an automatic scaffolding effect of the brain, these multilevel age-dependent changes can be counterbalanced by certain compensation mechanisms occurring with increasing age (Goh and Park, 2009; Park and Reuter-Lorenz, 2009; Reuter-Lorenz and Park, 2014). Hence, the elderly show bilateral recruitment of the two hemispheres even when task demand is low, as opposed to younger subjects who have more lateralized activity (Cabeza, 2002; Collins and Mohr, 2013). Furthermore, with aging, enhanced recruitment of broader networks involving parietal and temporal areas, besides postulated frontal hyper-recruitment, occurs (Calso et al., 2019). In the same vein, strengthened connectivity and sometimes neurogenesis, induced by new motor or cognitive tasks, e.g., by learning and practicing a musical instrument or social and intellectual engagements, have been proposed as aging compensation effects (McGinnis et al., 2011; Farina et al., 2017; Fukuda et al., 2018; Klöppel et al., 2018; Grassi et al., 2019).

### The Aging Musical Brain

Although previous research highlighted the need for multimodal investigations on elderly professional musicians to assess potential positive effects of musical activity during aging, neuroimaging studies have largely been lacking so far. Currently, it is known that musicians exhibit less age-related decline than non-musicians, especially in auditory processing functions such as detection of speech in noise or mistuned harmonics detection (Parbery-Clark et al., 2011; Zendel and Alain, 2012). In contrast, a large sample of professional symphony musicians experienced age-related performance decline because of physical, cognitive, sensory, and/or psychological problems with increasing age (Gembris and Heye, 2014). However, results from a more recent study on orchestral musicians suggest that playing a musical instrument may delay some age-related changes (Kenny et al., 2018). Based on these bodies of evidence, recent studies concluded that general functions could be preserved and compensatory cognitive processes could also be improved by musical practice at older age (Strong and Midden, 2018; Strong and Mast, 2019; for a meta-analysis, see Roman-Caballero et al. (2018)). Conclusively, musical practice could have beneficial effects on perception capacity, processing speed, inhibition, and attention. Moreover, the early onset and amount of musical activity might have a significant impact on these improvements (Strong and Midden, 2018; Strong and Mast, 2019). Therefore, high musical activity might act as a protective factor for cognitive domains that decline with age, e.g., by slowing down some deterioration processes or by working as a booster for cognitive domains that usually remain intact with age (Hanna-Pladdy and MacKay, 2011; Zendel and Alain, 2012; Fostick, 2019).

While the impact of musical activity on cognitive functions in the elderly has been extensively studied, little evidence exists on its relationship to cortical structure and function in elderly musicians. Most lifespan studies addressing music-dependent brain changes focused on middle-aged populations (20–66 years) and evaluated the effects of musical activity on healthy aging (Fauvel et al., 2013; Jäncke, 2013; Alain et al., 2019; Yurgil et al., 2020) or as an intervention tool for rehabilitation in neurodegenerative disorders (Sarkamo and Sihvonen, 2018; Kim and Yoo, 2019; Slattery et al., 2019; Feng et al., 2020; for a review, see Wan and Schlaug, 2010). However, studies on elderly professional musicians barely exist (Jäncke, 2013; Altenmüller and Furuya, 2016; Vaquero et al., 2016; Rogenmoser et al., 2018). Overall, these studies suggest that age-related brain degeneration could be counteracted by musical training, and that age at training onset might play a key role (Jäncke, 2013; Vaquero et al., 2016; Rogenmoser et al., 2018). For a corresponding review, see Altenmüller and Furuya (2016) and the ongoing longitudinal study by James et al. (2020) evaluating brain plasticity and cognitive benefits induced by musical training on elderly people. However, according to a recent review and meta-analysis, in general, not only musical activity but also professional occupation per se might shape the neural network and have beneficial effects in later life. In particular, functional activation of frontal-precentral regions and structural properties of STG and putamen could play a role as neural modulators in the “occupational neuroplasticity” process (Wu et al., 2020).

Based on existing evidence, this study aims to evaluate if and how structural and functional brain features that are specifically related to musicianship are associated with lifelong musical practice and if they are maintained in elderly musicians. To evaluate the potential age-related impact on cortical features in musicians, and as a surrogate for missing longitudinal design, a group of young music students was included for comparison. We hypothesize that despite common effects of brain aging, global functional and corresponding morphological properties of musicianship-relevant areas remain preserved with age in professional musicians.

## Materials and Methods

### Sample Description

Sixteen elderly professional musicians (OMs) and 15 elderly non-musicians (ONMs) who were matched for age, gender, and minimum years of education participated in the study (Table 1). To evaluate age-related effects, we also included a group of 16 young music students (YMs) during their first year of Bachelor studies at the Academy of Music Basel (Switzerland) who were matched for gender. The OMs and YMs were recruited from the Academy of Music, Basel and by recommendation. The OMs were previously working in the professional musical sector (e.g., music academy and music school) until or beyond retirement. The OMs and YMs were playing at least one main musical instrument (including singing), and most of the musicians also played several secondary musical instruments. All the YMs and 15 out of the 16 OMs were actively playing an instrument at the time of testing (Table 1). The ONMs were recruited by advertisements in local newspapers, talks in various local institutions, and by recommendation. They demonstrated a generally active lifestyle (e.g., dancing, sports, gaming, and music); however, they were not focused on musical activity. All the participants completed at least 12 years of school education, and all the elderly subjects completed at least 4 years of academic education. All the subjects were right-handed, as assessed by the modified/German version of the handedness questionnaire from Annett (1967).

**TABLE 1:** Sample description.

Variable	YMs (*N* = 16)	OMs (*N* = 16)	ONMs (*N* = 15)
Age	21.3 ± 2.4	76.6 ± 5.0	74.9 ± 3.4
	[18; 28]	[70; 85]	[71; 81]
Gender (m/f)	8/8	9/7	8/7
Starting age of musical activity (y)	5.31 ± 1.3	6.25 ± 0.8	(not specified)
	[4; 9]	[6; 9]	
Present intensity of musical activity (h/w)	30.31 ± 10.3 [12; 47]	13.53 ± 9.0 [0; 36]	3.07 ± 3.6 [0; 10]
Main musical instrument (played by *N* subjects)	piano (4)	piano (1)	piano (6)
	cello (1)	cello (3)	cello (1)
	violin (4)	violin (7)	violin (1)
	double bass (2)	double bass (1)	double bass (0)
	guitar (2)	guitar (0)	guitar (4)
	woodwind (2)	woodwind (2)	woodwind (1)
	drums (1)	drums (1)	drums (0)
	singing (0)	singing (1)	singing (0)
	no instrument (0)	no instrument (0)	no instrument (2)

*Data presented as: mean ± SD, [minimum; maximum], except for gender and main musical instrument. YMs, young musicians; OMs, old musicians; ONMs, old non-musicians; m, male; f, female; y, years; w, week; h, hours.*

Besides a demographic assessment to inquire for parameters age, gender, handedness, years of education, and present/former occupation, the participants were also interrogated with respect to their musical behavior during their lifetime. Hence, the following musical specific parameters were assessed: musical education/graduation stage (just for the musicians), played musical instruments (just for the musicians), intensity of musical activity, and starting age of musical activity. The musical activity parameters included only values for playing a musical instrument and singing, and no other passive musical activities. The intensity of musical activity was assessed separately for each musical instrument (including singing) and each decade of life, starting from the age of one until the time of this study. Based on these self-estimated data, we calculated several musicianship scores, which reflect the features duration, intensity and cumulative practice of professional musical behavior, across the subjects’ individual lifespan, as described in detail in the next section.

To probe the cognitive, mental, and medical health of the elderly subjects (OMs, ONMs), an extensive battery of neuropsychological tests, questionnaires, and initial phone screening was performed by a trained neuropsychologist. The cognitive tests assessed executive functions, interference management, and short-term working memory performance, and are displayed in Supplementary Table 1. A complete list of all the performed tests can also be found in Supplementary Table 10.

To detect potential age-related hearing loss, a hearing threshold assessment was performed on all the elderly participants using the audiometric toolbox AFC by Stephan D. Ewert (version 1.30).^1^ Binaural pure-tone audiograms were measured involving eight test frequencies (0.4, 0.8, 1, 2, 3, 4, 5, and 6 kHz) which were presented using Sennheiser HDA-200 audiometric headphones. Overall, the audiograms showed age-appropriate results, mostly including typical age-related hearing loss progressing within 3–6 kHz; for reference, see Rigters et al. (2018). To assess whether the elderly groups (OMs, ONMs) differed regarding their hearing thresholds, we performed independent *t*-tests on the results of each test frequency, revealing no significant differences among the groups (Supplementary Table 11 and Supplementary Figure 3). We adapted the sounds of our functional magnetic resonance imaging (fMRI) task accordingly based on the mean audiogram results to optimize listening quality for the elderly subjects during MRI measurement (see next section for details).

All the participants fulfilled the MRI compatibility criteria and had no history of neurological, psychiatric, or neurodegenerative disorders. All the participants gave their informed consent to participate in the study, which was approved by the local ethics committee of Basel, Switzerland.

### Evaluation of Musical Aptitude and Activity

To be able to characterize our groups with respect to musical activity, we performed an assessment of musical activity using questionnaires. We further used the musical activity parameters to assess the link between musicianship-specific behavior and functional and structural brain features. Furthermore, individual musical aptitude was assessed using the standardized Advanced Measures of Music Audiation test (AMMA), which evaluates audiation abilities that are essential to musical aptitude (Gordon, 1989).

**FIGURE 1:**
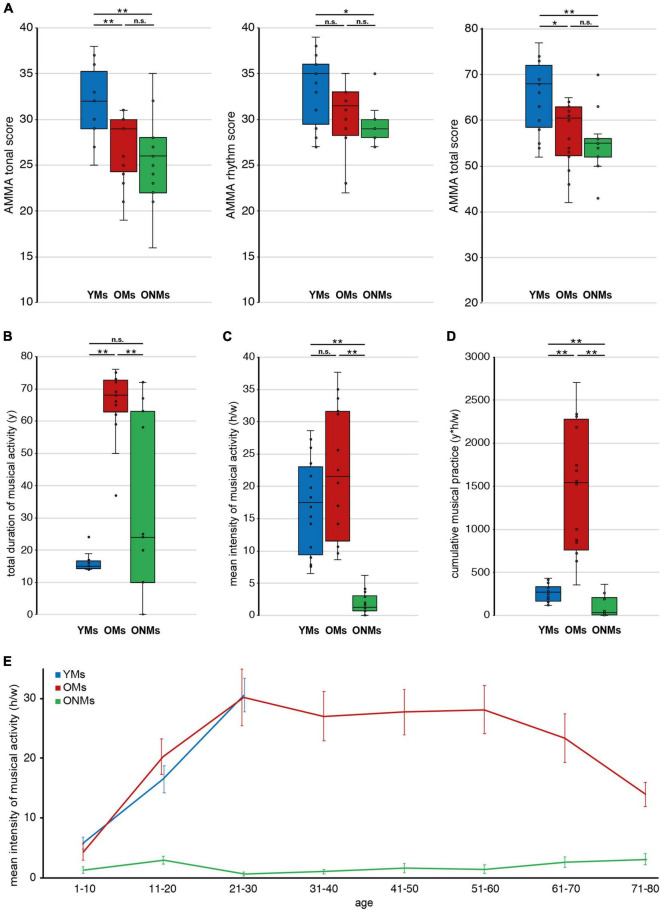
Musicianship-dependent scores. Average musicianship scores of young musicians (YMs), old musicians (OMs), and old non-musicians (ONMs): **(A)** tonal, rhythm, and total scores of the AMMA test; **(B)** total duration of musical activity (y) across the lifespan; **(C)** mean intensity of musical activity (h/w) presented for the lifespan; **(D)** cumulative musical practice (y*h/w) across the lifespan; **(E)** mean intensity of musical activity (h/w) presented for each decade separately, and error-bars represent SEM. **(A–D)** Whiskers in the boxplots represent data range. For more details, see corresponding Table 1. YMs, young musicians; OMs, old musicians; ONMs, old non-musicians; AMMA, Advanced Measures of Music Audiation test; y, years; h/w, hours/week; y*h/w, years*hours/week. **Significance level *p* < 0.001; *significance level *p* < 0.01; n.s., not significant.

The AMMA test is a reliable tool for evaluation of musical aptitude, as demonstrated within previous music-related studies (Schneider et al., 2002; Wengenroth et al., 2014; Leipold et al., 2021) and has proven good predictive and criterion validity (Gordon, 1990; Hanson, 2019). The AMMA test was performed, as obtained on https://www.giamusic.com/products/P-3372.cfm. In brief participants were exposed to a total of 30 short musical statements followed each by a musical answer. A musical statement consisted of a short piano sequence. After hearing a musical statement, the participants were exposed to another piano sequence, called musical answer. Now, the participants needed to decide if the musical answer was either equal or showed tonal or rhythm changes. The participants answered each decision on a sheet by crossing out “same,” “tonal change,” or “rhythm change.” The standardized test score calculation allows for the evaluation of the tone and rhythm scores separately in addition to the total score (Figure 1A).

Furthermore, several musicianship scores were calculated based on a subject’s individual self-estimations, and were defined as follows: (i) total duration of musical activity, measured in years (y): reflecting the summed years of actively playing a musical instrument and/or singing (Figure 1B); (ii) mean intensity of musical activity, measured in hours per week (h/w): reflecting the mean musical activity over a period of life. This score has been calculated by the addition of all average activity values within a decade as estimated for each stated musical instrument and/or singing (Figure 1E). For the evaluation of the mean activity across the individual lifespan, the activity values (per decade) have been also averaged over all decades of life (Figure 1C); (iii) cumulative musical practice, measured in year * hours per week (y*h/w): reflecting the musical practice accumulated over the lifetime, calculated based on the multiplication of the two preceding scores (Figure 1D). In addition, we also assessed the present intensity of musical activity (h/w), reflecting the present musical activity at the time of study screening, as well as the starting age of musical activity (y) for additional information, as presented in Table 1.

### Structural and Functional Data Acquisition

To evaluate potential structural and functional brain differences among the groups, structural and functional MRI of the brain was performed using a 3 Tesla MRI scanner (Verio, Siemens, Erlangen) with a 20-channel head and neck coil. High-resolution T1-weighted 3D MRI images were acquired using the following parameters: repetition time (TR) 1,570 ms, echo time (TE) 2.67 ms, 1 mm^3^ isotropic resolution, flip angle 9°, 192 contiguous sagittal slices, and matrix size 256 mm^2^ × 256 mm^2^. T2-weighted images were acquired using the following parameters: TR 8,000 ms, TE 77 ms, flip angle 9°, 40 transversal slices (parallel to the anterior-posterior commissure plane), slice thickness 3 mm, gap 6 mm, and matrix size 320 mm^2^ × 288 mm^2^. All the structural images were assessed by a trained neuroradiologist for potential pathologies. Whole-brain blood oxygenation level-dependent (BOLD) fMRI was acquired using a T2*-weighted gradient echo planar imaging (EPI) sequence with the following parameters: 38 oblique slices parallel to the AC plane, slice thickness 3 mm, gap 1 mm, TR 2,500 ms, and TE 30 ms.

During fMRI acquisition, the subjects performed a musical tone listening task and were instructed to attentively listen to presented sounds, as performed in our previous studies (Wengenroth et al., 2014; Benner et al., 2017). The paradigm had the following parameters: total task duration 4.25 min, block duration 20 s, individual tone duration within a block 500 ms (presented in a pseudo-randomized order with an inter-stimulus interval of 50 ms). The block design task included 6 tone and 7 baseline (rest/silent) blocks presented in alternating order and always started with a baseline block. The task tones consisted of five sampled instrumental sounds (piano, oboe, cello, trumpet, and guitar), covering the musically relevant fundamental tone range (0.1–2 kHz). With respect to audibility, the volume of the presented sounds was individually adjusted for each participant in between a level range of 70–80 dB_*SPL*_. Additionally, for the elderly participants only, the stimuli were generally optimized for better audibility, especially in higher frequencies using a soft shelving filter (+3 dB/oct at 3–6 kHz) to equalize the assessed average hearing loss. The motivation for this task was to use a stimulus that has been already approved in earlier studies and is known to stimulate a broad functional activation of the auditory cortex and related co-activations within the musical network (Zatorre, 2018).

### Whole Brain Analyses of Brain Structure

Most studies on elderly musicians evaluated effects of music on cognition and brain function (Altenmüller and Furuya, 2016; Roman-Caballero et al., 2018), but only a handful of studies evaluated the structure of the brain (Sluming et al., 2002; Abdul-Kareem et al., 2011; Vaquero et al., 2016; Rogenmoser et al., 2018). Moreover existing musician studies used mainly the voxel-based morphometry (VBM) method (Ashburner and Friston, 2000) to analyze the structure of the brain. It is however known, that this method entails some methodological pitfalls when specific regions or detailed structural differences are studied (e.g., voxels are classified as GM although they are positioned in WM, harder differentiation of deep in matter positioned sulci) (Hutton et al., 2009). Hence, in the present study we aimed to evaluate the structural changes occurring in elderly musician brains and compare them to non- and young musicians. Based on previous studies on young musicians, we hypothesize that structural properties belonging to the auditory network are maintained with age and preserved in elderly professional musicians despite the natural-occurring senescence. To evaluate this, we used a recent and validated state of the art structural analysis method, namely, surface-based analysis (SBM) (Dale et al., 1999; Fischl et al., 1999; Fischl and Dale, 2000). This method offers a more differentiated measure of brain structure by having as an output various GM metrics, i.e., thickness (THK), volume (VOL), surface area (SA), or gyrification. To this respect, recent longitudinal aging studies suggest separate evaluation of various GM metrics: first, because it is known that these metrics might exhibit different trajectories over lifetime, and, second, because it is still unclear which brain areas and metrics are sensitive to cognitive aging (Cox et al., 2018; Sele et al., 2021).

Moreover, how these various GM metrics are influenced by lifelong musical training, as encountered in elderly professional musicians, is still unknown. Hence, to obtain a broader and informative picture of existing neuroplastic mechanisms in elderly musicians, we evaluated the high resolution T1-weighted images by processing them using the standardized FreeSurfer 5.3 analysis pipeline^2^ as follows. As a *first step*, individual data of every subject were segmented and reconstructed into white surface and pial surface using the automatized recon-all command. In order to improve the segmentation of elderly brains, the acquired T2 images were used in the recon-all pipeline. Briefly, this pipeline implies motion correction, removal of non-brain tissue, automated Talairach (TAL) transformation, segmentation of the subcortical white and gray matters, intensity normalization, tessellation of the WM/GM boundary, automated topology correction, and surface deformation (Talairach and Tournoux, 1988; Sled et al., 1998; Dale et al., 1999; Fischl and Dale, 2000; Fischl et al., 2001; Fischl et al., 2002; Fischl et al., 2004; Segonne et al., 2004; Reuter et al., 2010). The reconstructed surfaces for each participant were then visually inspected, and minor defects were manually corrected as recommended by the software guidelines. As a *second step*, image processing and computation of the three GM metrics were performed: THK, VOL, and SA. Note that the SA is white area, as automatically given by FreeSurfer. Then, the implemented framework of the general linear modeling (GLM) (glm_fit command) was applied, and THK, VOL, and SA values were computed at the level of each vertex, for each hemisphere separately, and in each participant of the three groups (YMs, OMs, and ONMs). As a final step, the following group statistics were performed: (i) to evaluate general atrophy effects, we determined whole-brain differences in the three GM metrics between musicians (YMs vs. OMs) and between the elderly groups (OMs vs. ONMs); (ii) to evaluate how exactly these metrics are influenced by age or music and if there is difference in this effect between groups, slope difference analysis of the GM metrics in the aforementioned two group contrasts was performed and analyzed for the following variables: age, mean intensity of musical activity, and AMMA scores; (iii) to evaluate if, how, and which brain area and GM metric are associated with age and music, correlations of all the three GM metrics with age, mean intensity of musical activity, and AMMA scores were performed by groupwise structural covariance analyses. To correct for multiple testing, we performed Monte Carlo simulations with 10,000 iterations in order to identify significant contiguous clusters (*p* < 0.05) of vertex-wise group differences.

### Individual Analysis of Auditory Cortex Morphology

The segmentation-based shape analysis of individual AC was performed for reasons of comparability with our previous studies showing increased AC size and gyrification patterns in musicians (Schneider et al., 2002; Schneider et al., 2009; Wengenroth et al., 2014; Benner et al., 2017). The aim of this study was to evaluate if similarly extended gyrification of the AC could be found in elderly professional musicians and, hence, is maintained with age despite neurodegeneration. Therefore, individual semiautomatic cortical segmentation and 3D reconstruction of the right and left STG were performed on all the subjects using the BrainVoyager QX 2.8 software (Brain Innovation, Maastricht, The Netherlands). This procedure has been described previously in more detail (Benner et al., 2017; Zoellner et al., 2019). Briefly, this method involves segmentation of individual STG based on preprocessed T1-weighted images and subsequent 3D reconstruction of the extracted STG and its subparts, including anterior STG, planum temporale (PT), HG and possible adjacent posterior gyral duplications. Identification of distinct STG subparts was performed by applying standard definitions of anatomical landmarks (Rademacher et al., 2001; Penhune et al., 2003; Wong et al., 2008) and by established structural criteria of AC (Schneider et al., 2005b; Wengenroth et al., 2014; Benner et al., 2017). Besides visualization of individual STG reconstructions of every subject, further outputs are structural characteristics of different HG morphotypes and their occurrence frequencies. For simplification, in this study, we defined only two types of AC morphology, namely, single HG and duplicated HG. The latter encompasses several subtypes of posterior HG duplication previously described in Benner et al. (2017) and exemplified in Supplementary Figure 1B. We hypothesize that musicianship-specific morphotypes of the AC can also be observed in elderly professional musicians even at older age.

### Functional Data Analyses

Functional MRI (fMRI) data were analyzed using the BrainVoyager QX 2.8 software (Brain Innovation, Maastricht, The Netherlands). The automated and standardized preprocessing pipeline included slice time correction, motion correction, temporal filtering, and transformation to TAL space. Additionally, the pipeline required two manual steps, i.e., individual overlay of functional images on structural MRI images and individual definition of reference points for spatial normalization. Groupwise functional activation maps of tone vs. baseline were calculated in every group based on voxelwise calculation of BOLD signal and using linear cross-correlations (GLM). To assess how musicians differ from non-musicians in their functional activation maps elicited by auditory stimulation during fMRI, group contrast activation maps were calculated (YMs vs. OMs, OMs vs. ONMs, and YMs vs. ONMs) by computing a cluster-wise correction for multiple comparisons (FDR, *p* < 0.01). Furthermore, to exactly study the effects of age and music on the observed functional brain changes, we assessed the association of functional activations (BOLD signals) with age in the group of musicians (YMs and OMs together) and the association of functional activation with each of the musical parameters (AMMA scores, mean intensity of musical activity, and cumulative musical practice) in the combined group of the elderly (OMs and ONMs together). To this end, we performed functional random effects (RFX) group analyses (as described in BrainVoyager v22.0) (Roebroeck et al., 2005) with age or musicianship scores as covariates and using a cluster level correction threshold (*p* < 0.02). Overall, from these functional results, we hypothesize that musicians, independent of age, show much broader functional activation in areas that are part of the musicianship-related network, as described in Rauschecker (2018). We hypothesize further that age does have an influence on these functional activation patterns to a different degree in musicians and non-musicians.

### Region of Interest Analyses

As observed in our previous studies in adult musicians, certain brain areas, i.e., the AC, are structurally and functionally correlated to musical performance and aptitude in professional musicians (Schneider et al., 2005b; Wengenroth et al., 2014; Benner et al., 2017). However, at the group level, these effects are mostly obscured because of high inter-individual and inter-hemispheric variability. Taking this into account, the aim of our computed region of interest (ROI) analysis was to test if similar patterns can also be observed in the elderly population of musicians. Therefore, we used areas known to be of relevance in musicians and are comparable to our previous work, and areas that evolved to be significant in the present sample. We tested further to which extent these areas are able to differentiate the groups from each other and how they relate to music-specific parameters and age. Hence, we hypothesize that, independent of age, areas belonging to the AC and music-related integrative areas, such as parietal and inferior frontal areas, might be of relevance with respect to structure and function in professional musicians. The ROI level analysis was performed using IBM SPSS Statistics (Version 27.0; IBM Corp, Armonk, NY, United States).

#### Region of Interest Preselection

The structural ROIs involved regions from both FreeSurfer atlases, i.e., Desikan (gross division), and the Destrieux atlas (fine division). Therefore, ROI average values from all the metrics (THK, VOL, and SA) and from each of the two hemispheres were considered. The ROIs that significantly distinguished the elderly groups (according to an independent sample *t*-test) were selected for further discriminant analysis. Additionally, average values per hemisphere of the GM metrics, THK, VOL, and SA (from the FreeSurfer aseg atlas), were included. The functional ROIs used for further statistical analysis implied the beta values of regions that showed significant BOLD activation differences during the attentive listening fMRI task (tone vs. baseline) in the group contrasts (OMs vs. ONMs, YMs vs. OMs, and YMs vs. ONMs).

#### Discriminant Function Analysis

We used the ROIs that significantly distinguished the elderly groups (independent *t*-tests) and performed four discriminant analyses. This has advantages over interpretation of results of various independent *t*-tests. Discriminant analysis takes relationships among variables into account, makes corrections for unnecessary multiple comparisons, and provides information on which variables discriminate the groups best. The AMMA scores (tonal, rhythm, and total) and musicianship scores (total duration of musical activity, mean intensity of musical activity, and cumulative musical practice) were non-normally distributed, which is why we performed Kruskal-Wallis tests that were followed by Whitney-Mann group comparisons. For all other measures, we conducted parametric analyses. The selected structural and functional ROIs were hereby used as predictor variables in the analysis. A discriminant function analysis with structural ROIs was performed at a two-group and at a three-group level. The two-group level discriminant analysis implied a pairwise comparison of the groups (YMs vs. OMs, OMs vs. ONMs, and YMs vs. ONMs) and aimed to assess how well the ROIs distinguished among these groups. The three-group level discriminant analysis implied all the three groups at once and aimed to assess how the selected ROIs separate age-dependent (elderly vs. young) and musicianship-dependent (musicians vs. non-musicians) aspects from each other. To be able to judge the importance of the discriminant coefficients to the standardized discriminant functions, an arbitrary cutoff value of >0.3 was used. A discriminant function analysis with functional ROIs was performed at the two-group level by pairwise comparisons between groups.

#### Correlation Analysis

To evaluate the association with musicianship, Pearson correlation analyses (based on normally distributed data) were performed between the abovementioned structural ROIs and musicianship scores (AMMA, total musical activity duration, and cumulative score of musical practice).

Furthermore, a Chi-quadrat analysis was conducted to provide information on the relationship between HG morphotype frequencies and musical status (musicians vs. non-musicians).

## Results

### Differences in Cognition and Musicianship Scores

Results of the initial neuropsychological tests, assessed for the elderly subjects to control for cognitive performance, revealed no significant differences between OMs and ONMs after Bonferroni correction (Supplementary Table 1). In the AMMA test results (Figure 1A and Supplementary Table 2), the YMs differed significantly from the ONMs in all three scores (YMs vs. ONMs, tonal and total *p* < 0.001, rhythm *p* < 0.01) and from the OMs in the tonal and total scores (YMs vs. OMs, tonal *p* < 0.001, total *p* < 0.01) but not in the rhythm score after Bonferroni correction (rhythm *p* < 0.05). None of the AMMA scores did significantly differ between the elderly groups (OMs vs. ONMs). Looking for a relationship with age, all the AMMA scores were negatively correlated with age across all groups (whole sample, tonal *r* = -0.53, rhythm *r* = -0.46, total *r* = -0.52, all *p* (two-tailed) < 0.01). Regarding musical activity, the OMs showed significantly higher scores in total duration of musical activity and cumulative musical practice than both the YMs and the ONMs (*p* < 0.01). Furthermore, the OMs showed significantly higher values in the mean intensity of musical activity (across lifetime) only compared to the ONMs (*p* < 0.01) but not to YMs (Figures 1B–D and Supplementary Table 2).

### Differences in Whole Brain Structure

**FIGURE 2:**
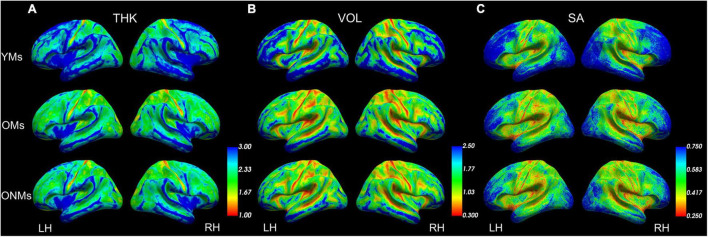
Average structural metrics. Group averaged maps for **(A)** thickness, **(B)** volume, and **(C)** surface area projected on group-averaged inflated pial surfaces. Color scales present smaller values in red and higher values in blue (set up using FreeView). See Supplementary Table 3 for more details. YMs, young musicians; OMs, old musicians; ONMs, old non-musicians; THK, thickness; VOL, volume; SA, surface area; LH/RH, left hemisphere/right hemisphere.

**FIGURE 3:**
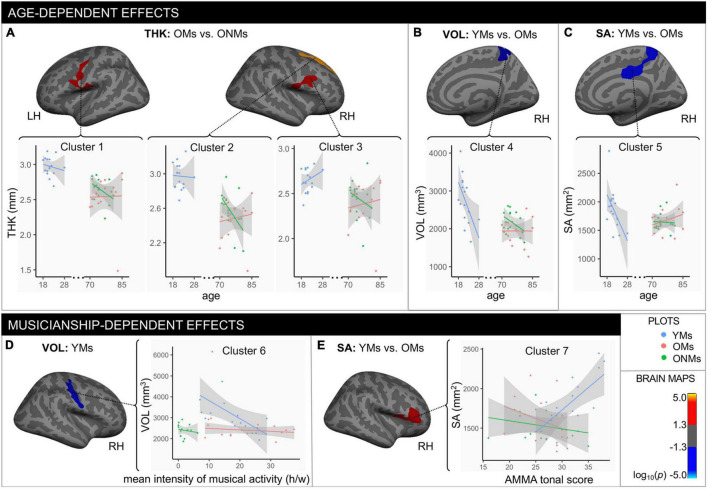
Age- and musicianship-dependent structural differences. Age-dependent effects: clusters that showed significant changes with age in **(A)** thickness, **(B)** volume, and **(C)** surface area between the specific groups (output from FreeSurfer age-slope difference analysis). Musicianship-dependent effects: **(D)** cluster that showed significant association between the mean intensity of musical activity (h/w) and volume in the YMs (results from covariance analysis); **(E)** cluster that showed significant surface area differences between the YMs and the OMs with changes in AMMA tonal score (output from FreeSurfer AMMA-slope difference analysis). All depicted clusters evolved from the general linear model (GLM) analysis. The multiple comparison correction method is Monte Carlo 10,000 simulations with a setup clustering threshold of log10(*p*-value), which was set at *p* = 0.05; hence the color bar indicates positive (red/yellow) vs. negative (blue/turquoise) significant values. Cluster details can be found in Table 2. Note that the depicted brain clusters only refer to specific group differences found in the GLM (age-slope analysis: **A–C**; covariance analysis: **D**; AMMA-slope analysis: **E**), while the scatterplots include values for all the three groups. Scatterplots that included all the three groups were only inserted for descriptive purposes and better readability of the data and were not part of the GLM analysis. The gray area around the lines represents the 95% confidence level interval for predictions from a linear model (“lm”). YMs, young musicians; OMs, old musicians; ONMs, old non-musicians; THK, thickness in mm; VOL, volume in mm^3^; SA, surface area in mm^2^; LH/RH, left hemisphere/right hemisphere; AMMA, Advanced Measures of Music Audiation test; y, years, h/w, hours/week.

The whole brain structural group analysis revealed that, on average, all GM metric values (THK, VOL, and SA) were significantly higher in the YMs than in both elderly groups (OMs and ONMs) (Figure 2 and Supplementary Table 3). The peak differences between the musicians (YMs vs. OMs) were located in the STG, precuneus, lateral/medial-OFC, fusiform gyrus, postcentral, and superior-frontal areas (for details see Supplementary Table 4). There were no significant whole brain structural differences between the two elderly groups (OMs vs. ONMs). However, when evaluating for the GM metrics as a function of age, the elderly groups (OMs vs. ONMs) showed significant age-dependent slope differences in THK values in three clusters (Figure 3A and Table 2). Specifically, THK values in these clusters were decreased with increasing age in the ONMs when compared to the OMs, in which THK values remained stable or increased with increasing age (see scatterplots in Figure 3A). According to FreeSurfer Desikan atlas annotations, the significant clusters encompassed parts of the following brain areas: cluster 2 (*p* < 0.01) right superior-frontal and rostral-anterior-cingulate (rACC) areas, clusters 1 and 3 (*p* < 0.05) bilateral pre-/post/para-central and IFG pars-opercularis areas, and cluster 3, additionally, right caudal- and rostral-middle-frontal areas. Furthermore, the musician groups (YMs vs. OMs) showed age-dependent slope differences in VOL and SA values in two clusters (Figures 3B,C and Table 2). Specifically, VOL and SA values in these clusters decreased with increasing age in the YMs compared to the OMs, in which the VOL and SA values remained stable or increased (see scatterplots in Figures 3B,C). Both significant clusters contained parts of right para-/post-central, superior parietal (SPL), and precuneus areas (*p* < 0.05), and cluster 5, additionally, parts of the right posterior cingulate (PCC) area.

**TABLE 2:** Age- and musicianship-dependent effects.

ClusterNr	Annot	Hemi	Max	VtxMax	Size (mm^2^)	MNI X	MNI Y	MNI Z	CWP	CWPLow	CWPHi	NVtxs

Age-dependent effects – slope differences												
**Thickness: OMs vs ONMs**												
Cluster 1	Postcentral	lh	3.649	102628	1172.12	–56.9	–11.7	13.9	0.00818	0.00659	0.00978	2787
Cluster 2	Superiorfrontal	rh	3.810	80289	2383.19	20.7	21.3	50.7	0.00020	0.00000	0.00040	4295
Cluster 3	Postcentral	rh	4.257	83566	869.56	57.7	–6.6	10.8	0.04391	0.04019	0.04762	2000
**Volume: YMs vs OMs**												
Cluster 4	Precuneus	rh	−5.073	99936	903.46	13.1	–47.2	56.3	0.04780	0.04510	0.05050	2253
**Surface area: YMs vs OMs**												
Cluster 5	Precuneus	rh	–3.976	81709	1644.75	10.4	–48.0	67.7	0.01673	0.01435	0.01911	4235

**Musicianship-dependent effects**												

**Correlation: volume and mean intensity of musical activity in YMs**												
Cluster 6	Pre/post/sub – central G/S	rh	–3.209	81907	1290.30	48.1	–13.2	28.5	0.00590	0.00490	0.00690	2981
**AMMA slope differences: YMs vs OMs**												
Cluster 7	Parstriangularis	rh	3.224	25840	1425.19	52.6	30.5	2.8	0.04371	0.03999	0.04742	2705

*Clusters that showed significant age- and musicianship-dependent differences between the groups (YMs, OMs, and ONMs) in specific GM metrics. The clusters evolved from the general linear model analysis (see Figure 3) after cluster-wise correction for multiple comparisons by Monte Carlo simulations (10,000 steps, threshold p = 0.05). Hemi, hemisphere; lh/rh, left hemisphere/right hemisphere; Cluster Nr, cluster number as shown in Figure 3; Annot, annotation of cluster peak; Max, indicates maximum – log10 (p-value) in the cluster; VtxMax, vertex number at maximum; Size, surface area (in mm^2^) of cluster, MNI X/Y/Z, MNI space (MNI305) coordinates of the maximum; CWP, clusterwise p-value; CWPLow and CWPHi, 90% confidence interval for CWP; NVtxs, number of vertices in cluster.*

Concerning musicianship-dependent effects, the YMs showed a significant association of their mean intensity of musical activity with VOL values in a cluster involving right pre-/post- and subcentral areas (cluster 6: *p* < 0.01, Figure 3D and Table 2). Furthermore, both musician groups (YMs vs. OMs) showed significant AMMA score-dependent slope differences in SA values in a cluster containing parts of right IFG pars-opercularis/triangularis and rostral-middle-frontal areas (cluster 7: *p* < 0.05, Figure 3E). Specifically, the SA values in this cluster increased with increasing AMMA score in the YMs compared to the OMs, in which the SA values remained stable or decreased (see scatterplots in Figure 3E).

### Individual Differences in Shape and Gyrification of Auditory Cortex

**FIGURE 4:**
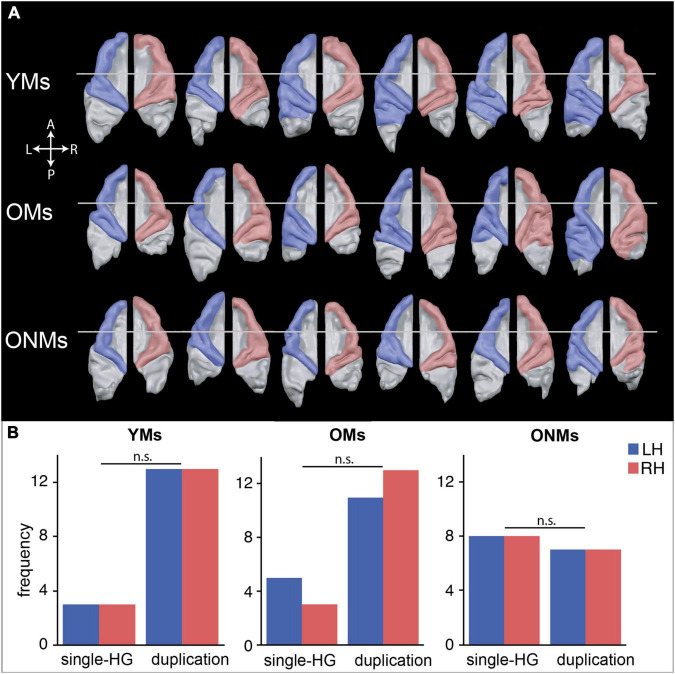
Inter-individual shape differences of the auditory cortex (AC). **(A)** Subsample of Supplementary Figure 1 demonstrating representative AC surfaces of six exemplary participants, and depicting the characteristic individual shapes of the left (blue) and right (red) AC within each group. Further AC reconstructions of all the subjects are presented in Supplementary Figure 1. **(B)** Observed frequencies of the two defined AC types, single and duplicated Heschl’s gyrus (HG), are counted separately per hemisphere. A more detailed definition of HG morphotypes can be found in Supplementary Figure 1B. LH/RH, left hemisphere/right hemisphere; YMs, young musicians; OMs, old musicians; ONMs, old non-musicians; HG, Heschl’s gyrus; n.s., not significant (contrast).

Semiautomated segmentation and surface reconstruction of the STG in each of the subjects revealed high inter-individual and inter-hemispheric variability of AC morphology in the three groups (Figure 4A). Both musician groups (YMs and OMs) displayed increased frequency of duplication-HG type compared to single-HG type, in contrast to the ONMs who presented an approximately equal amount of the two HG types in both hemispheres, with a tendency towards more single-HG types (Figure 4B). We performed a chi-square test, which showed that duplication-HG types were found in 81% of the YMs (for both hemispheres) and in 69% (for LH) and 81% (for RH) of the OMs, whereas only 47% of the ONMs had duplication-HG types (for both hemispheres). However, because of the small sample size, these differences were not statistically significant [*X*^2^(2) = 4.21, *p* > 0.05].

### Differences in Whole Brain Function

**FIGURE 5:**
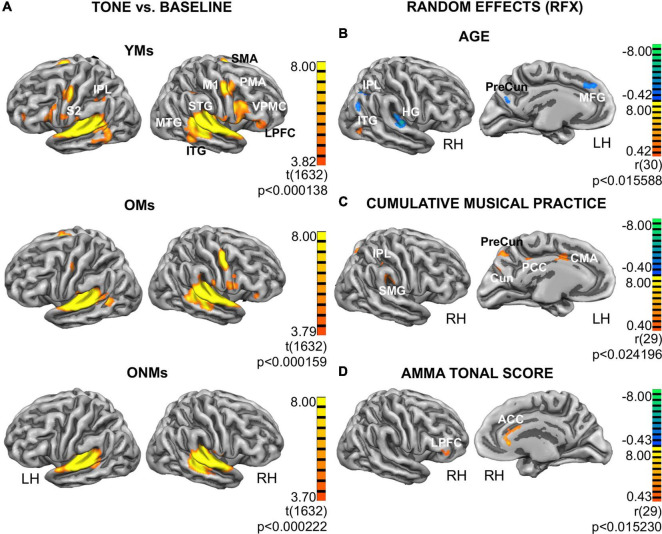
Functional group activations and correlation analyses. **(A)** Functional results for the attentive tone listening functional MRI (fMRI) task: activation maps from contrast tone listening vs. baseline depicted for each group (YMs, OMs, and ONMs). The results are presented at the threshold FDR (*p* < 0.01) and projected on the inflated pial surface of a standard average brain. Red-yellow represents voxels showing increased activity during task (task > baseline). Additional group contrast results can be found in the Supplementary Tables 5–7. **(B–D)** Results from the random effects (RFX) group analysis. Only views with significant clusters are presented. Depicted are functional clusters correlating with: **(B)** age of musicians (YMs and OMs); **(C)** cumulative musical practice (y*h/w) in the elderly (OMs and ONMs); **(D)** AMMA tonal score in the elderly (OMs and ONMs). The results have been corrected at cluster level (*p* < 0.02). Red-yellow represents positive and blue-green negative correlations, respectively. For more details, see Table 3. YMs, young musicians; OMs, old musicians; ONMs, old non-musicians; LH/RH, left hemisphere/right hemisphere; RFX, random effects group analysis; AMMA, Advanced Measures of Music Audiation test; M1, primary motor cortex; SMA, supplementary motor area; PMA, premotor area; S2, secondary somatosensory cortex; IPL, inferior parietal lobule; SMG, supramarginal gyrus; HG, Heschl’s gyrus; STG, superior temporal gyrus; MTG, middle temporal gyrus; ITG, inferior temporal gyrus; VPMC, ventral premotor cortex; LPFC, lateral prefrontal cortex; MFG, middle frontal gyrus; PreCun, Precuneus; Cun, Cuneus; ACC, anterior cingulate cortex; PCC, posterior cingulate cortex; CMA, cingulate motor area.

The attentive tone listening fMRI task elicited activation of bilateral primary and secondary auditory areas in all the three groups. The activated regions involved large aspects of the STG including HG, anterior temporal transverse sulcus (aHS), superior temporal sulcus (STS), and planum polare (PP) and PT. The musicians (YMs and OMs) co-activated a much broader network of auditory association areas, primary and secondary motor areas, and prefrontal and parietal regions (Figure 5A). However, BOLD signals in these musicianship-specific co-activations were significantly lower in the OMs than in the YMs (Supplementary Table 5). To assess age- and musicianship-dependent effects on the BOLD-signals, random effects (RFX) correlation analyses were performed using age and the musicianship scores as covariates (Figure 5B). Correlation of BOLD signals with age in the musician groups (YMs and OMs) revealed that activation in the following regions significantly decreased with increasing age: HG, inferior parietal lobule (IPL), precuneus, medial frontal gyrus (MFG), and cerebellum (Figure 5B, upper panel). This age-dependent negative correlation was highest in HG [LH *r* = -0.53, *p* (two-tailed) < 0.02; RH *r* = -0.51, *p* (two-tailed) = < 0.02; see Table 3 for details]. An age-dependent positive correlation was calculated in the inferior temporal gyrus (ITG). Correlation of BOLD-signal with two musicianship scores (mean intensity of musical activity and cumulative musical practice) in the elderly groups (OMs and ONMs) revealed that activation in the following regions was significantly increased with increasing musicianship scores: supramarginal gyrus (SMG), IPL, precuneus, cuneus, cingulate motor area (CMA), and posterior cingulate cortex (PCC) (Figure 5C and Table 3). Furthermore, AMMA tonal score correlated positively with the BOLD signals in the ACC and right lateral prefrontal cortex (LPFC) in the elderly sample (OMs and ONMs) (Figure 5D and Table 3).

**TABLE 3:** Random effects (RFX) functional results.

ROI	Hemi	*r*	TAL X	TAL Y	TAL Z	N Voxel
**YMs & OMs with covariate age**						
Heschl’s Gyrus	lh	–0.53	–50.04	–25.36	8.59	3507
Heschl’s Gyrus	rh	–0.51	51.85	–20.30	9.69	2389
Inferior parietal lobule	lh	–0.50	–38.57	–69.08	35.98	2934
Cerebellum	rh	–0.49	18.59	–40.71	–25.33	713
Inferior parietal lobule	rh	–0.49	36.84	–59.02	41.40	1540
Cerebellum	lh	–0.49	–37.22	–68.26	–35.82	2111
Cerebellum	lh	–0.48	–11.95	–81.37	–34.63	945
Cerebellum	rh	–0.48	21.93	–70.03	–35.31	3308
Inferior parietal lobule	lh	–0.48	–45.70	–46.33	48.12	871
Precentral gyrus (M1)	lh	–0.47	–40.26	–35.54	52.96	2602
Precentral gyrus (M1)	lh	–0.47	–37.51	–30.16	55.47	1723
Precuneus	lh	–0.47	–5.20	–62.73	20.46	653
Medial frontal gyrus	lh	–0.47	–4.25	31.24	39.35	813
Inferior temporal gyrus	rh	0.46	40.83	–66.46	–3.94	616
**OMs & ONMs with covariate mean intensity of musical activity (h/w)**						
Supramarginal gyrus	rh	0.47	53.92	–29.07	29.40	832
Premotor area	rh	0.47	49.54	4.32	15.66	581
Insula	lh	0.46	–28.56	–1.69	4.79	838
Cingulate motor area	lh	0.46	–1.82	2.90	38.52	1496
Precuneus	lh	0.46	–5.27	–67.37	41.52	506
Insula	rh	0.45	40.71	7.07	1.83	736
Cuneus	lh	0.45	–4.15	–74.77	23.43	230
OMs & ONMs with covariate cumulative musical practice (y*h/w)						
Supramarginal gyrus	rh	0.46	54.27	–28.60	28.65	857
Precuneus	lh	0.45	–5.31	–66.06	44.47	753
Premotor area	rh	0.45	46.71	3.27	21.81	1100
Insula	lh	0.45	–29.17	–2.75	4.97	638
Cingulate motor area-cingulate gyrus frontal	lh	0.45	–0.32	2.87	38.34	1697
Inferior parietal lobule	rh	0.44	40.03	–40.84	41.15	1015
Insula	rh	0.44	40.15	8.38	0.23	748
Cingulate gyrus dorsal	rh	0.44	–1.70	–36.52	33.09	1714
Cuneus	lh	0.44	–4.30	–74.42	25.96	410
**OMs & ONMs with covariate AMMA tonal score**						
Anterior cingulate cortex	rh	0.48	1.80	25.70	17.83	1497
Posterior cingulate cortex	lh	0.47	–9.56	–41.99	19.56	396
Lateral prefrontal cortex	rh	0.46	49.73	37.46	9.11	403

Significant clusters evolved from the random effects (RFX) group analyses (see Figures 5B–D), i.e., with covariate age and in the elderly groups (OMs and ONMs), as well as with covariates mean intensity of musical activity (h/w), cumulative musical practice (y*h/w), and AMMA tonal score in the musician groups (YMs and OMs), respectively. The cluster threshold was set at p < 0.02. Hemi, hemisphere; lh/rh, left hemisphere/right hemisphere; ROI, region of interest; r, correlation score; TAL X/Y/Z, mean Talairach coordinates; N voxel, number of voxels in cluster; YMs, young musicians; OMs, old musicians; ONMs, old non-musicians; y, year; h, hour; w, week; AMMA, Advanced Measures of Music Audiation test.

### Structural Regions of Interest Discriminating the Groups

**FIGURE 6:**
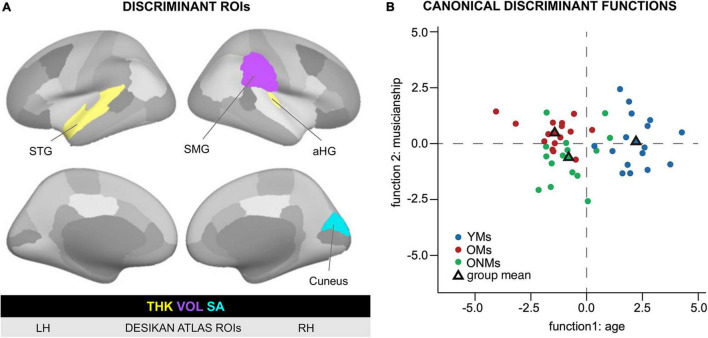
Discriminant function analysis based on structural ROIs. ROIs that separated the groups based on structural metrics. **(A)** Visualization of the ROIs that resulted to be significant in the discriminant function analysis. The left and right hemispheres as well as lateral and medial views are presented based on the FreeSurfer Desikan atlas. The color code (yellow = THK, violet = VOL, and turquoise = SA) indicates the respective GM metric in the specific ROI. **(B)** Discriminant function analysis plot visualizing the separation between the young and old subjects on function 1 and musicians and non-musicians on function 2 based on Desikan atlas structural ROIs. For more details, see Table 4. Based on the structural ROIs in panel A, the first discriminant function discriminated best between the OMs/ONMs and the YMs, i.e., young vs. old, while the second function differentiated the OMs from the ONMs, i.e., musicians vs. non-musicians. YMs, young musicians; OMs, old musicians; ONMs, old non-musicians; LH/RH, left hemisphere/right hemisphere; THK, thickness; VOL, volume; SA, surface area; ROI, region of interest; group mean, mean variate score for each group called centroid in the discriminant analysis; STG, superior temporal gyrus; SMG, supramarginal gyrus; aHG, anterior Heschl’s gyrus.

The average values of THK, VOL, and SA per hemisphere, which were used as predictor variables in the pairwise discriminant analysis, separated YMs vs. OMs, as well as YMs vs. ONMs with 100% discrimination accuracy (both with *p* < 0.01, Table 4). However, these predictor variables could not separate the two elderly groups from each other (OMs vs. ONMs). In addition, all these average GM metrics were highly negatively associated with age in the whole group of subjects: VOL Supramarginal gyrus (RH: *r* = −0.65), THK Superior temporal gyrus (LH: *r* = −0.78), THK Heschl’s gyrus (RH: *r* = −0.56), and SA Cuneus (RH: *r* = −0.56), all *p* (two-tailed) < 0.001. Correlations remain significant after Bonferroni correction for multiple testing. The ROI preselection analysis based on the Desikan atlas (see section “Region of Interest Analyses”) revealed four ROIs that significantly distinguished OMs vs. ONMs, i.e., STG (LH and THK), SMG (RH and VOL), aHG (RH and THK), and cuneus (RH and SA) (Figure 6A). These ROIs were used further in both two-group and three-group level discriminant analyses.

**TABLE 4:** Significant structural ROIs of the discriminant function analysis.

Analysis level 2: Differential	Predicting brain area (descending relevance)	Hemi	Meas	Wilk’s	CCA	df	*p*	Hit rate	*r*
**Global measures**									
YMs vs.OMs	Mean GM	rh	VOL	0.14	0.93	6	0.00	100	0.69
		lh	VOL						0.68
	Mean GM	rh	THK						0.57
		lh	THK						0.53
	Mean WM	lh	SA						0.33
		rh	SA						0.30
YMs vs. ONMs	Mean GM	rh	VOL	0.12	0.94	6	0.00	100	0.61
		lh	VOL						0.60
	Mean GM	rh	THK						0.51
		lh	THK						0.51
	Mean WM	lh	SA						0.31
		rh	SA						0.30
OMs vs. ONMs	Same variables as above			0.79		6	n.s.		
**FreeSurfer Desikan Atlas ROIs**									
YMs vs. OMs	Superior temporal gyrus	lh	THK	0.21	0.89	4	0.00	96.9	0.86
	Supramarginal gyrus	rh	VOL						0.49
	Heschl’s gyrus	rh	THK						0.45
	Cuneus	rh	SA						0.33
YMs vs. ONMs	Superior temporal gyrus	lh	THK	0.28	0.85	4	0.00	90.3	0.71
	Supramarginal gyrus	rh	VOL						0.52
	Cuneus	rh	SA						0.50
	Heschl’s gyrus	rh	THK						0.36
OMs vs. ONMs	Superior temporal gyrus	lh	THK	0.67	0.57	4	0.03	77.4	0.71
	Cuneus	rh	SA						–0.54
	Heschl’s gyrus	rh	THK						0.37
	Supramarginal gyrus	rh	VOL						0.15

*Results of the two-group level discriminant function analysis of the structural ROIs: upper panel, ROIs containing the average GM per hemisphere and metric (thickness and volume surface area); lower panel, specific ROIs from the FreeSurfer Desikan atlas. These clusters are also depicted in Figure 6. Hemi, hemisphere; lh/rh, left hemisphere/right hemisphere; YMs, young musicians; OMs, old musicians; ONMs, old non-musicians; THK, thickness; VOL, volume; SA, surface area; ROI, region of interest; r, contribution to discriminant function; Hit rate, discrimination accuracy; Meas, measure; Wilk’s, Wilk’s Lambda test; CCA, canonical correlation analysis; df, degrees of freedom.*

#### Two-Group Level Discriminant Analysis Results

Altogether, the four preselected ROIs had high discrimination accuracy in the pairwise comparisons of the groups, i.e., the ROIs could separate with 77.4% accuracy the OMs from the ONMs, with 96.9% the YMs from the OMs, and with 90.3% the YMs from the ONMs (Table 4). The STG had the highest contribution to the discriminant function in all the three comparisons. However, when comparing the individual contribution of each ROI to the discriminant function, the SMG had the lowest rate (*r* = 0.15) in separating the OMs vs. ONMs.

#### Three-Group Level Discriminant Analysis Results

In this analysis, all the three groups entered simultaneously the discriminant analysis function based on the previously performed two-group analysis of the Desikan atlas ROIs. The analysis resulted in two discriminant functions (Figure 6B). Discriminant function 1 had a canonical correlation of 0.86; thus, it was strongly related to group membership. The canonical correlation of discriminant function 2 was 0.41, which illustrated that it was rather moderately related to group membership and had a statistically significant chi-square test [*X*^2^ (3) = 7.91, *p* < 0.05]. Furthermore, the combined predictive value of both discriminant functions 1 and 2 was statistically significant [*X*^2^ (8) = 64.5, *p* < 0.01]. The prediction of group membership showed that 78% of the variance in discriminant scores was due to between-group differences. In total, 83% of the participants were correctly classified into the respective groups (YMs, OMs, and ONMs) based only on the preselected ROIs. STG, SMG, and aHG had the largest discriminant coefficients for the standardized discriminant function 1 (cutoff 0.3, see section “Methods” for details). In contrast, the cuneus was the only relevant predictor for discriminant function 2.

Overall, the YMs had higher, but not significantly higher values in all the four ROIs than the other two groups (OMs and ONMs). Of note is that only in the cuneus the ONMs had smaller values than the OMs, while in the other three ROIs (STG, SMG, and aHG), the difference was in the opposite direction, ONMs > OMs. The discriminant analysis based on the finer cortex division of the Destrieux atlas had overlapping results and is, therefore, only presented briefly here (for details see Supplementary Figure 2 and Supplementary Table 9). Hence, i.e., additionally to the STG subregions (PP, PT, SMG, and temporal pole), precuneus, dorsal posterior cingulate cortex (dPCC), and sulcus intermedius primus of Jensen contributed highest to the discriminant function. The discrimination accuracy of these regions was comparable to the Desikan atlas, i.e., 93.5–100% depending on group contrast.

### Functional Regions of Interest Discriminating the Groups

The pairwise discriminant analysis (two-group level) revealed that the YMs could be separated from the OMs with 100% accuracy based on the BOLD signals in the cerebellum, IFG, and MFG. The two elderly groups (OMs and ONMs) could be separated from each other with 96.8% accuracy based on the following regions: middle occipital gyrus, MTG, HG, and STG. Of note is that here, the HG and STG had a negative load on the discriminant function as opposed to the MTG and occipital regions. YMs vs. ONMs could be separated with 96.8% accuracy by cerebellum and STG regions (Table 5). Since three-group level analyses for the functional ROIs did not provide significant differences, we included only two-group level results.

**TABLE 5:** Significant functional ROIs of the discriminant function analysis.

fMRI contrast	Predicting brain area (descending relevance)	Hemi	Wilk’s	CCA	df	*p*	Hit rate	*r*
YMs vs. OMs	Cerebellum	lh	0.3	0.84	3	0.00	100	0.65
	Inferior frontal gyrus	rh						0.51
	Medial frontal gyrus	lh						0.48
YMs vs. ONMs	Cerebellum	lh	0.28	0.85	3	0.00	96.8	0.75
	Cerebellum	rh						0.60
	Superior temporal gyrus	lh						0.44
OMs vs. ONMs	Middle occipital gyrus	rh	0.29	0.84	4	0.00	96.8	0.49
	Middle temporal gyrus	lh						0.45
	Heschl’s gyrus	rh						–0.36
	Superior temporal gyrus	lh						–0.34

*Results of the two-group level discriminant function analysis of functional ROIs. The functional ROIs are based on the beta-values of individual regions from group contrasts. Hemi, hemisphere; lh/rh, left hemisphere/right hemisphere; YMs, young musicians; OMs, old musicians; ONMs, old non-musicians; r, contribution to discriminant function; Hit rate, discrimination accuracy; Wilks’, Wilks Lambda test; CCA, canonical correlation analysis; df, degrees of freedom; ROI, region of interest.*

## Discussion

### The Musical Profile of Elderly Musicians Resembles That of Young Musicians

In this study, we assessed structural and functional cortical features and their association with musical activity and aptitude in elderly professional musicians with lifelong musical practice using a multiparametric approach. The effect of lifelong intensive musical activity on the aging brain was evaluated by comparing these cortical features in the elderly musicians (OMs) to those in the young musicians (YMs) and those in the elderly non-musicians (ONMs). While recent studies have focused mainly on musical (Kenny et al., 2018) and cognitive performances of elderly musicians (Roman-Caballero et al., 2018; Strong and Midden, 2018; Strong and Mast, 2019), there is a serious demand for corresponding neuroimaging studies. The goal of this study was to fill this gap and, hence, present results that offer new evidence on the influence of lifetime musical activity on various cortical features of the aging brain, and provide new hints on the potential neuroprotective role that music might entail. As expected, when comparing the lifelong musical activity profiles of our study groups, the OMs were much more like the YMs (Figures 1C,E), but they significantly differed from the ONMs, who had an active but mostly music-unrelated lifestyle (Figures 1B,D).

Interestingly, with respect to the AMMA scores, the two elderly groups did not differ significantly (Figure 1A); however, there was a consistent trend toward higher AMMA scores in the musicians for all three AMMA scores (tonal, rhythm, and total), which was clearest in the tonal score. Taking into account that the AMMA test was initially designed for rather young populations and, hence, was developed and validated more often in college students (both music majors and non-majors), high school students, and junior high school students (Gordon, 1990), our results suggest that the sensitivity to distinguish the elderly groups from each other is slightly less accurate. With respect to the distribution of the AMMA scores over lifetime, previous studies have shown an increase from birth to adulthood remaining in a stabilized state across adult life (Gordon, 2004; Walters, 2010). However, whether musical aptitude scores remain unchanged in elderly musicians has not been addressed so far and needs more systematic longitudinal studies that include adults. Nevertheless, a recent meta-analytic review proved again the criterion validity of the AMMA test (Hanson, 2019), hence, showing a certain entitlement that the AMMA test is of value, at least, in music education research. Future studies on elderly musicians should, however, take into account this factor and elaborate in more detail on its distribution at older age.

### Elderly Musicians Preserve Structural Cortical Features Despite Senescence

The widespread whole brain atrophy effects we observed in all the elderly participants in the evaluated metrics (THK, VOL, and SA) (Figure 2) were not surprising, and these results confirm previous studies that have shown a patchwork of regionally atrophied vs. spared areas with aging (Lemaitre et al., 2012; Choi et al., 2019). As the whole-brain group difference contrast analysis did not reveal any significant differences between the two elderly groups (OMs and ONMs) in any of the three evaluated GM metrics, only significant group differences between the young and the old (see Figure 2) suggest stronger atrophy than musicianship-dependent effects. However, several ROIs did show significant structural changes with increasing age in the elderly when specifically analyzing age slope differences between the respective groups in the various metrics. Hence, parts of the ventrolateral PFC and premotor cortex showed stable or even increasing THK values in the OMs with increasing age as opposed to the ONMs (Figure 3A). According to the “last in first out” aging theory, which assumes that regions that mature last are first to degenerate, we know that frontal areas are prone to degenerate first (Jackson, 1958; Dempster, 1992; Raz et al., 2005; Calso et al., 2019; Ebaid and Crewther, 2020). Hence, the results of this study show rather favorable brain plasticity of frontal areas in the musicians and suggest that “regions at risk” might be protected by the effect of musicianship. Therefore, we also confirm previous studies, which showed that music-making might have age-decelerating effects on the brain. However, a recent study also indicated that too intense musical engagement might diminish these effects (Rogenmoser et al., 2018).

Furthermore, despite widespread brain atrophy, as well as inter-individual and inter-hemispheric variability, the shape of the AC was more alike in both musician groups, independent of age, and differed from that of the AC of the non-musicians (Figures 4A,B). Hence, the encountered musicianship-specific morphology of the AC in the OMs was similar to previously reported findings in young and adult musicians. According to these studies, the prevailing increased size and gyrification of HG might be associated with musicianship (Schneider et al., 2002; Benner et al., 2017), pitch perception preference (Schneider et al., 2005b; Wengenroth et al., 2014), and musical instrument preference (Schneider et al., 2005a). Even though the difference in AC gyrification between our groups did not reach statistical significance, most probably because of the small sample size and high inter-individual variability of AC morphology, the distribution of the observed HG duplication frequencies corresponded with the musicianship-specific HG features described in our previous studies. Hence, we assume that musicianship related anatomical features, such as extended gyrification within the AC, are detectable in musicians even at older age. However, to what extent these anatomical features are preserved needs further clarification in studies with larger sample sizes. Nevertheless, we computed further discriminant function statistics using a ROI-based approach and demonstrated that the two elderly groups (OMs and ONMs) could be separated with 77.4% discrimination accuracy based on the structural features of areas within the AC, i.e., STG, aHG, and SMG (Figure 6A and Table 4). Musicianship seems to imprint the structure of main auditory-associated areas to such an extent that solely on this imprint, elderly musicians can be distinguished from non-musicians at older age. Of note is that increased gyrification of the AC was also found in expert phoneticians (Golestani and Pallier, 2007; Golestani et al., 2011) and in individuals with Williams syndrome (a genetic disorder with strong affinity to music and sound) (Wengenroth et al., 2010), pointing to a genetic basis of AC macro-anatomy.

### Anterior and Posterior Brain Regions Differentiate Young and Old Musicians

Besides the widespread global atrophy-related differences between the young and old subjects, structural differences in specific ROIs were observed with increasing age between the two musician groups (YMs and OMs). Hence, in precuneus/PCC regions, the OMs had stable VOL and SA values, while in the YMs values were decreasing with age. This may be in part explained by the higher inter-individual structural variability in these regions in the YMs; however, it could also indicate age-dependent changes in precuneus/PCC regions of the musicians (Figures 3B,C). These posterior association areas are known to structurally decrease with age, and it has been assumed that this decrease is compensated by functional hyperactivation of frontal areas with aging (Davis et al., 2008; Hafkemeijer et al., 2014; Davis et al., 2019). Structural and functional alterations in these areas have also been linked to development of neurodegenerative disorders in the elderly (Klaassens et al., 2017; Yokoi et al., 2018). In musicians, this region has been attributed to be a separation hub for complex auditory scenes (e.g., melody, rhythm, and harmony), as well as an integration point of emotions to the respective scenes before information is further translated to the motor system for musical performance (Spada et al., 2014; Tabei, 2015; Tanaka and Kirino, 2016, 2017). Hence, the effect of age encountered among musicians in this relevant separation and integration hub might, in part, explain the observed musical performance degradation in elderly musicians (Kenny et al., 2018). Moreover, the structural decrease in the precuneus/PCC of the YMs might be linked to a developmental maturational process of these brain areas, and to the neuroplastic impact that intense musical training at this specific age might have on the brain (Raz, 2009; Hafkemeijer et al., 2014; Jockwitz et al., 2017; Klaassens et al., 2017; Yokoi et al., 2018; Jockwitz et al., 2019).

Interestingly, the structural properties of these and adjacent areas (precuneus/PCC, cuneus) together with AC regions discriminated the musician groups with 97% accuracy (Table 4 and Figure 6), as well as the elderly groups with 77% accuracy. This indicates that these areas are prone to both age and musicianship-dependent effects. These results complement the findings by Tanaka and Kirino (2017) who found a comparably high discrimination accuracy (87%) of similar areas (precuneus/PC and auditory areas) between musicians and non-musicians. However, these latter findings were based on the functional connectivity of the mentioned regions as opposed to our results, which were based on structural properties. Overall, the emerging evidence on the structural and functional discrimination power of posterior brain areas suggests that these areas might be of relevance in the aging process of professional musicians.

Furthermore, the YMs and OMs showed musicianship-dependent structural differences. Hence, decrease of VOL values in motor areas was associated with increased intensity of musical activity in the YMs (Figure 3D). This reduction effect on gray matter might be driven by highly automatized motor skills that musicians obtain by long-term musical activity, also shown in previous studies (Groussard et al., 2014; James et al., 2014; Wu et al., 2020). In addition, increase of SA values in the right IFG was associated with increased AMMA tonal score in the YMs but not in the OMs (Figure 3E). This observation fits well to the AMMA score differences found at a behavioral level between the same two groups (Figure 1A). Despite the high importance of its left counterpart (Broca), the right IFG is a relevant hub for musicians (Sluming et al., 2002; Abdul-Kareem et al., 2011). It is part of an optimized ventral auditory stream in musicians and has been linked to detection of fine-grained incongruities in music and to musical aptitude (Tourville and Guenther, 2011; James et al., 2014; LaCroix et al., 2015; Rauschecker, 2018; Palomar-Garcia et al., 2020; Leipold et al., 2021).

### Elderly Musicians Maintain the Extended Functional Activation Network

Both the YMs and the OMs demonstrated a broader functional activation network, especially in the right hemisphere, when only listening to simple instrumental tones, while the ONMs showed a locally more confined activation around the AC (Figure 5A). This musicianship-specific activation network encompassing frontotemporal, sensorimotor, and parietal areas has been described in previous studies (Jäncke, 2013; Zatorre and Salimpoor, 2013). In musicians, co-activation of these brain areas, in addition to the AC, is in line with the dual-stream model of auditory processing and sensorimotor integration, which incorporates two pathways, a dorsal stream integrating audio-motor processing and a ventral stream involving tone identification (Gordon et al., 2018; Rauschecker, 2018).

Overall, our functional results complement previous findings providing new evidence of a preserved musicianship-specific functional network in elderly musicians despite senescence. Nevertheless, age-dependent effects were detectable at a whole-brain level by a weaker but region-wise similar activation in the OMs than in the YMs. Specifically, age-dependent decrease in functional activation was prominent in HG, the precuneus, IPL, and MFG, whereas age-dependent increase in functional activation was observed in the ITG, as revealed by the correlation analyses (Figure 5B). Note that similar areas also showed age-dependent structural changes in the elderly (Figure 3). Hence, this structural and functional overlap strengthens the anterior-posterior shift hypothesis, which postulates posterior decline to be coupled with frontal hyperactivation in the elderly (Davis et al., 2008, 2019). Interestingly, some of these age-dependent differences mirrored musicianship-dependent effects when comparing the OMs to the ONMs. Specifically, increased functional activation in the OMs related to cumulative musical practice and intensity of musical activity was detected in the precuneus and IPL and in secondary motor (CMA) and auditory (SMG) regions (Figure 5C). In addition, increased functional activation in the OMs related to AMMA tonal score was detected in the right IFG (LPFC) and ACC (Figure 5D and Table 3). Note again that the structure of similar regions also showed age- and musicianship-dependent effects (Figure 3 and Table 2). This suggests that structural and functional features in auditory and non-auditory regions are preserved in elderly professional musicians, and puts emphasis on musical activity as a neuroprotective factor. The relevance of these areas in active playing and listening has been replicated by other studies and shown to be influenced by occupational neuroplasticity (Bangert and Schlaug, 2006; Baumann et al., 2007; Jockwitz et al., 2017; Cheung et al., 2018; Jockwitz et al., 2019; Leipold et al., 2019; Wu et al., 2020; Leipold et al., 2021). Furthermore, the high accuracy of 97% with which musicians could be discriminated from non-musicians only based on functional data also at older age (Table 5) is in line with previous studies (Tanaka and Kirino, 2016, 2017). Particularly, the right IFG was one of the most relevant regions for discriminating the YMs from the OMs, whereas temporal regions discriminated the OMs from the ONMs.

Overall, this study demonstrates that structural differences between musicians and non-musicians were mostly detectable at ROI-based level, and that functional differences were already prominent at the whole-brain level. This confirms recent results from Leipold et al. (2021) who showed that musical training particularly affects cortical function rather than structure.

### Functional and Structural Lateralization Effects: A Matter of Music?

The results of this study demonstrate characteristic right hemispheric functional activation in professional musicians (Figure 5) and right hemispheric age- and musicianship-dependent effects of GM density (Figure 3). Overall, this functional and structural overlap in the right hemisphere encompasses core auditory processing areas such as the AC (in function), and areas for musical integration such as premotor, parietal, inferior frontal, and cingulate areas. However, interpretation of these asymmetries is not straightforward, as studies evaluating the effects of musical experience on hemispheric lateralization bring rather mixed results. Hence, the right hemispheric dominance for tonal processing in musicians has previously been related to dominant spectral or absolute pitch perception (Schneider et al., 2005b; Wengenroth et al., 2014). Evidence from structural studies showed increased GM concentration and VOL in the right AC of musicians (Bermudez et al., 2009; Palomar-Garcia et al., 2017), and right lateralized effects on relevant white matter tracts, such as the arcuate fasciculus, have been observed, i.e., higher fractional anisotropy in the right fasciculus of musicians (Halwani et al., 2011). As opposed to that, other studies reported bilateral activations during musical feature processing (Ono et al., 2011), or left lateralized activation during regular rhythm perception in musicians (Limb, 2006). Overall, in this respect, more evaluations need to be performed to be able to draw solid conclusions on potential right lateralization of music in the brain. Moreover, taking into account the results from a recent study by Villar-Rodriguez et al. (2020) reporting higher probability for atypical language lateralization in left-handed musicians, a detailed sample description is needed to be able to compare the results to other studies and, hence, avoid confounding factors.

### Methodological Limitations Influencing Data Interpretability

When interpreting structural results, one should be aware that most previous musician studies used a voxel-based approach (VBM) to evaluate GM, while our study applied the recent state-of-the-art surface-based method (SBM), as used by FreeSurfer, which overcomes some methodological pitfalls of VBM (Dale et al., 1999; Fischl et al., 1999; Fischl and Dale, 2000; Hutton et al., 2009). One further advantage when using SBM is that it offers a much more differentiated measure of GM. Hence, when comparing our results to those of previous studies, one should account for these methodological differences. However, the exact association of various GM metrics as well as the extent to which these are affected in different brain regions is still unclear (Dickerson et al., 2009; Lemaitre et al., 2012; Hogstrom et al., 2013; Choi et al., 2019). Conclusively, the question remains if a specific metric or a combination of those helps in elucidating age-dependent effects in musicians.

## Conclusion and Future Studies

According to the presented data, elderly musicians maintain the extended functional and structural cortical features of the known musicianship-related network despite senescence. Therefore, the preserved functional features were evident at the whole-brain level, and the structural features were more prominent at the ROI level. Hence, future studies that will investigate neural correlates in elderly musicians are encouraged to evaluate these features in a longitudinal design, tackling the questions, i.e., to which extent these specific neural features of the musician’s brain relate to predisposition and practice dependent factors, how they exactly relate to musician specific skills, and if they are protected from neurodegeneration with aging.

## Data Availability Statement

The raw data supporting the conclusions of this article will be made available by the authors, without undue reservation.

## Ethics Statement

The studies involving human participants were reviewed and approved by the local ethics committee of Basel, Switzerland. The patients/participants provided their written informed consent to participate in this study.

## Author Contributions

MB, CB, and JR designed the study. OR-O, JB, and JR performed all data analysis. OR-O, JB, MB, JR, and PS discussed the data interpretation. OR-O, JB, JR, MC, and MB wrote sections of the manuscript. MC performed the advanced statistical analysis. OR-O and JB performed the main literature research. CB, JR, EH, and JB acquired the data and performed participants recruitment. MB, CS, RK, and PS supervised the study and reviewed the manuscript. All authors revised the manuscript critically for important intellectual content and approved the submitted version.

## Conflict of Interest

The authors declare that the research was conducted in the absence of any commercial or financial relationships that could be construed as a potential conflict of interest.

## Publisher’s Note

All claims expressed in this article are solely those of the authors and do not necessarily represent those of their affiliated organizations, or those of the publisher, the editors and the reviewers. Any product that may be evaluated in this article, or claim that may be made by its manufacturer, is not guaranteed or endorsed by the publisher.

## Footnotes

1
medi.uni-oldenburg.de/afc/

2
http://surfer.nmr.mgh.harvard.edu/
