# Supplementary material for: Musicianship-Related Structural and Functional Cortical Features Are Preserved in Elderly Musicians
Source: Front Aging Neurosci. 2022 Mar 25;14:807971. doi: 10.3389/fnagi.2022.807971 (PMC8990841; doi:10.3389/fnagi.2022.807971)
Supplement: Supplementary file 1 [file Data_Sheet_1.pdf]

Supplementary Material

Supplementary Figures

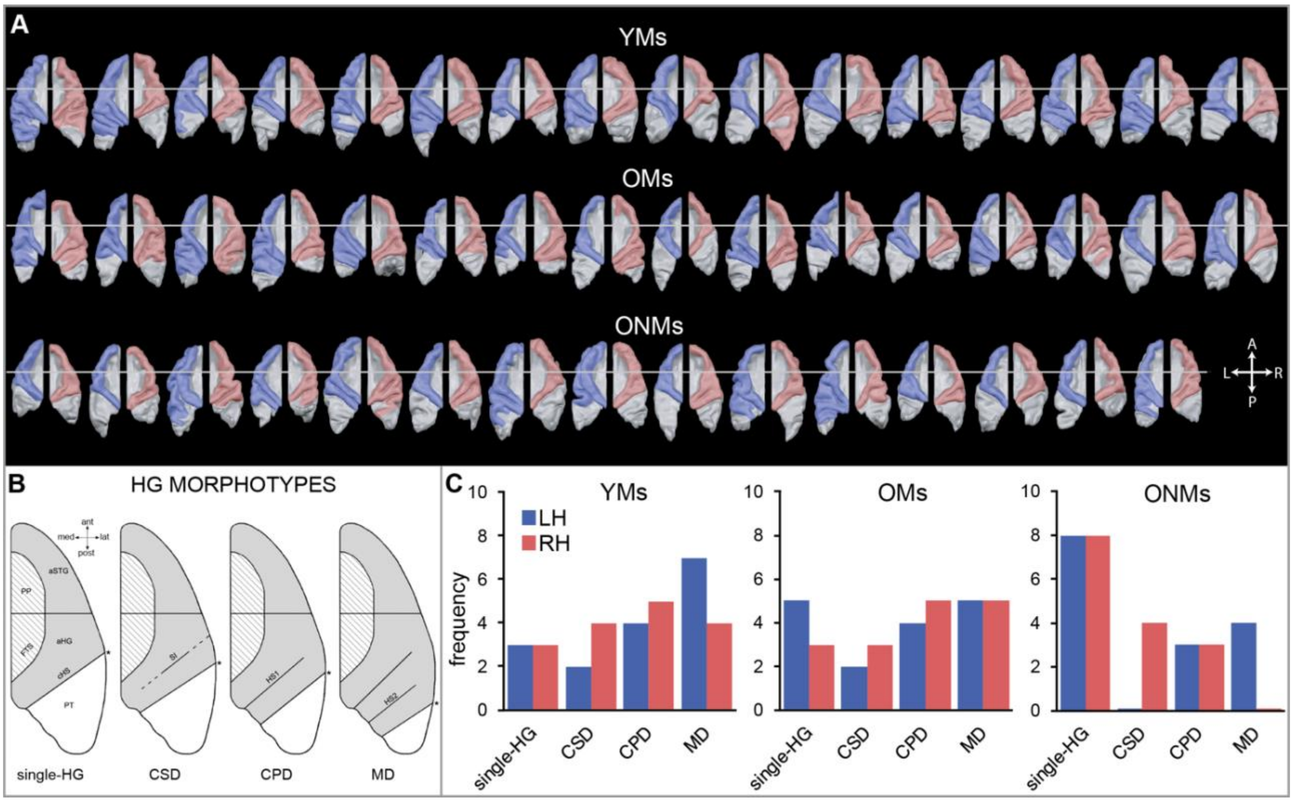

Supplementary Figure 1: Shape differences of individual auditory cortices
Inter-individual morphology of auditory cortices (AC): (**A**) 3D reconstructions of individual ACs are shown for each group for all participants, demonstrating the characteristic morphology of left (blue) and right (red) AC within superior temporal gyrus (STG). (**B**) Schematic representation of the most common morphotypes of Heschl's gyrus (HG). We considered CSD, CPD, MD morphotypes together in one category named HG duplication. Anatomical landmarks are depicted within STG. The lateral end of first complete Heschl's sulcus (cHS) is marked with asterisk (*). Supplementary Figure 1B adapted from Benner et al. (2017), with permission from Springer Nature. (**C**) Distribution of the observed HG morphotypes counted per hemisphere. LH/RH - left and right hemisphere, YMs - young musicians, OMs - old musicians, ONMs - old non-musicians, CSD - common stem duplication, CPD - complete posterior duplication, MD - multiple duplication (in reference to Benner et al. 2017), ant - anterior, post - posterior, med - medial, lat - lateral, aHG - anterior Heschl's gyrus, PP - planum polare, PT - planum temporale, FTS - first transverse sulcus, SI - sulcus intermedius, HS1 - first Heschl's sulcus, HS2 - second Heschl's sulcus, cHS - first complete Heschl's sulcus.

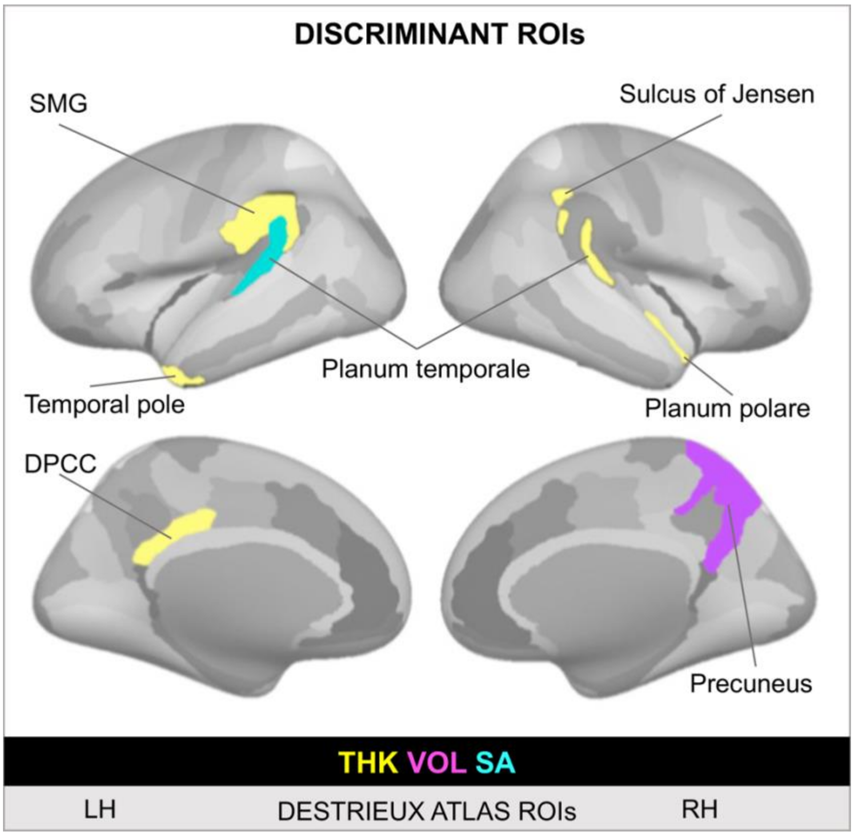

Supplementary Figure 2: Discriminant function analysis based on structural ROIs (Destrieux atlas)
ROIs which separated the groups based on the structural metrics. A visualization of the ROIs which resulted significant in the discriminant function analysis (for more details see **Supp.Tab.9**). The left and right hemispheres as well as the lateral and medial views are presented based on the FreeSurfer Destrieux atlas. The color code (yellow=THK, violet=VOL, turquoise=SA) indicates the respective GM metric of the specific ROI. LH/RH - left and right hemisphere, THK - thickness, VOL - volume, SA - surface area, ROI - region of interest, SMG - supramarginal gyrus, DPCC - dorsal posterior cingulate cortex.

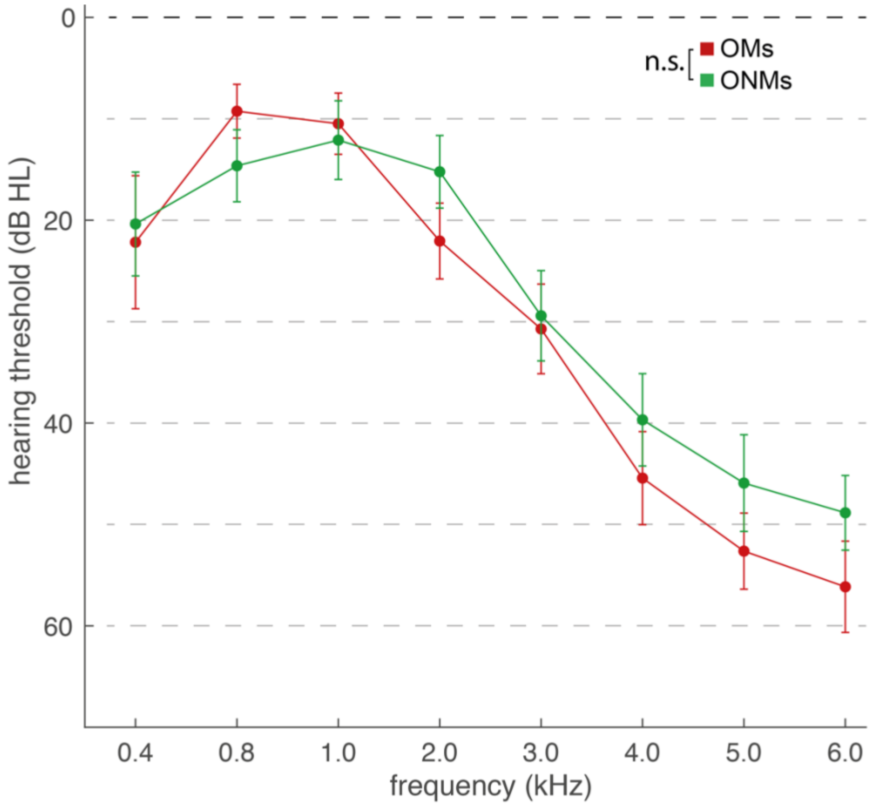

Supplementary Figure 3: Audiogram assessment of elderly participants
Initial assessed hearing thresholds of OMs and ONMs demonstrate the typical age-related hearing loss in elderly participants. Error-bars represent standard deviation. OMs - old musicians, ONMs - old non-musicians, n.s. – not significant (group contrast)

Supplementary Tables

Variable	Measures	OMs mean	OMs SEM	ONMs mean	ONMs SEM	t	df	p
**MOCA**	Cognitive assessment	27.50	0.438	27.73	0.419	-0.384	29	0.704
**TMT (A and B)**	Executive functions	44.38	2.448	42.00	3.080	0.608	29	0.548
	Task switching	108.94	6.460	112.00	16.410	-0.178	29	0.860
**VST**	Interference	14.63	0.861	13.00	0.655	1.488	29	0.148
	Inhibition	18.13	0.774	16.33	0.622	1.789	29	0.084
	Versions A. B. C	28.38	2.123	24.93	1.311	1.358	29	0.185
**Raven**		36.06	1.619	39.27	1.948	-1.271	29	0.214
**WMS – R**								
Verbal Span	Verbal and spatial Short-term memory and working memory	7.06	0.520	6.80	0.341	0.416	29	0.680
Forward / Backward		6.56	0.408	6.27	0.371	0.534	29	0.597
Visual Span		7.31	0.395	8.00	0.447	-1.156	29	0.257
Forward / Backward *		8.25	0.359	6.67	0.465	2.715	29	0.011

Supplementary Table 1: Neuropsychological tests performed in elderly
OMs - old musicians, ONMs - old non-musicians, MOCA - Montreal Cognitive Assessment Scale, TMT - Trial Making Test, VST - Stroop Color-Word Test, Raven - Progressive Matrices, WMS-R - Wechsler Memory Scale revised (German version). * - significant group differences. After correction of multiple testing the Forward / Backward visual span does not remain significantly different. All tests were performed two-tailed.

**Contrast**	**p**	**sig**	**effect size**	**z**	**U**
**AMMA total score**					
Kruskal Wallis Test for all 3 groups: H(2) = 13.04, p < 0.001					
**YMs vs OMs**	0.004	p < 0.01	"-0.49	-2.79	54.00
**OMs vs ONMs**	0.276	n.s.	"-0.20	-1.11	92.00
**YMs vs ONMs**	0.001	p < 0.001	"-0.58	-3.23	38.50
YMs (Md = 68; SEM= 1.91); OMs (Md = 60.5; SEM= 1.77); ONMs (Md = 55; SEM= 1.56)					
**AMMA tonal score**					
Kruskal Wallis Test for all 3 groups: H(2) = 15.48, p < 0.001					
**YMs vs OMs**	0.001	p < 0.001	-0.55	-3.09	46.50
**OMs vs ONMs**	0.234	n.s.	-0.22	-1.21	89.50
**YMs vs ONMs**	0.000	p < 0.001	-0.63	-3.49	32.00
YMs (Md = 32; SEM= 0.97); OMs (Md = 29; SEM= 0.93); ONMs (Md = 26; SEM= 1.18)					
**AMMA rhythm score**					
Kruskal Wallis Test for all 3 groups: H(2) = 9.67, p < 0.008					
**YMs vs OMs**	0.017	p < 0.05*	"-0.42	-2.37	65.50
**OMs vs ONMs**	0.156	n.s.	"-0.26	-1.44	84.00
**YMs vs ONMs**	0.007	p < 0.01	"-0.48	-2.67	53.00
YMs (Md = 35; SEM= 0.98); OMs (Md = 31.5; SEM= 0.95); ONMs (Md = 29; SEM= 0.49)					
**mean intensity of musical activity (h/w)**					
Kruskal Wallis Test for all 3 groups: H(2) = 30.93, p < 0.001					
**YMs vs OMs**	0.180	n.s.	-0.24	-1.36	92.00
**OMs vs ONMs**	0.000	p < 0.001	-0.85	-4.76	0.00
**YMs vs ONMs**	0.000	p < 0.001	-0.85	-4.76	0.00
YMs (Md = 27.5; SEM= 2.58); OMs (Md = 12; SEM= 2.32); ONMs (Md = 2; SEM= 0.93)					
**total duration of musical activity (y)**					
Kruskal Wallis Test for all 3 groups: H(2) = 26.35, p < 0.001					
**YMs vs OMs**	0.000	p < 0.001	-0.86	-4.85	0.00
**OMs vs ONMs**	0.000	p < 0.001	-0.61	-3.37	35.00
**YMs vs ONMs**	0.038	p < 0.05 *	-0.37	-2.07	68.00
YMs (Md = 15; SEM= 0.64); OMs (Md = 68; SEM= 2.53); ONMs (Md = 24 ; SEM= 6.98)					
**cumulative musical practice (y*h/w)**					
Kruskal Wallis Test for all 3 groups: H(2) = 34.86, p < 0.001					
**YMs vs OMs**	0.000	p < 0.001	-0.83	-4.71	3.00
**OMs vs ONMs**	0.000	p < 0.001	-0.85	-4.71	1.00
**YMs vs ONMs**	0.001	p < 0.001	-0.59	-3.28	37.00
YMs (Md = 270.5; SEM= 24.14); OMs (Md = 186.52; SEM= 1.77); ONMs (Md = 30 ; SEM= 32.16)					

Supplementary Table 2: Differences in musical scores

Statistical comparisons of musical scores between the individual groups. The table contains the results from the analysis of variances using the non-parametric Kruskal-Wallis-Test for all three groups and then the Mann-Whitney-U Test for the separate group comparisons corrected for multiple comparisons by Bonferroni procedure, which controls FDR threshold level. The group comparisons were corrected for multiple testing by applying a Bonferroni correction. All tests were performed two-tailed. YMs - young musicians, OMs - old musicians, ONMs - old non-musicians, Md - median, * - not significant after Bonferroni correction (p < 0.05)

	THICKNESS	VOLUME	SURFACE AREA
	**Left Hemisphere**		
**YMs**	2.64 ± 0.11	271875.30 ± 24163.37	90754.59 ± 8752.96
	[2.43 ; 2.83]	[224524.31 ; 310589.95]	[77262.50 ; 107274.00]
**OMs**	2.29 ± 0.14	201945.26 ± 17558.51	78565.48 ± 6463.08
	[1.82 ; 2.44]	[159441.23 ; 225067.12]	[68652.70 ; 88484.60]
**ONMs**	2.36 ± 0.10	205258.82 ± 18122.62	77474.24 ± 7676.14
	[2.08 ; 2.47]	[170406.01 ; 243963.53]	[62529.00 ; 90607.60]
	**Right Hemisphere**		
**YMs**	2.63 ± 0.10	271837.90 ± 23601.00	91009.04 ± 8869.32
	[2.42 ; 2.78]	[225022.98 ; 313685.46]	[76842.70 ; 107117.00]
**OMs**	2.29 ± 0.17	202287.55 ± 19266.87	79450.48 ± 6951.35
	[1.77 ; 2.44]	[156745.63 ; 231477.71]	[69771.50 ; 90044.10]
**ONMs**	2.36 ± 0.11	205328.63 ± 17666.56	78049.55 ± 7655.53
	[2.08 ; 2.51]	[171246.03 ; 240244.12]	[63565.20 ; 91531.80]

Data is presented as: mean ± standard deviation, [minimum ; maximum]

Supplementary Table 3: Average structural metrics

Average thickness (in mm), volume (in mm^3), and surface area (in mm^2) in young musicians (YMs), old musicians (OMs) and old non-musicians (ONMs) for the left and right hemisphere respectively.

Annot	Hemi	Max	VtxMax	Size (mm^2)	MNI X	MNI Y	MNI Z	CWP	CWPLow	CWPHi	NVtxs
**THICKNESS**											
precuneus	lh	10.749	21428	55676.93	-20.9	-64.4	12.4	0.00020	0.00000	0.00040	110058
superiortemporal	rh	11.888	79157	56541.65	47.9	0.4	-18.4	0.00020	0.00000	0.00040	113056
**VOLUME**											
lateralorbitofrontal	lh	12.692	110280	66056.49	-13.6	53.9	-16.4	0.00020	0.00000	0.00040	128691
medialorbitofrontal	rh	14.575	4937	66900.47	7.0	51.7	-20.8	0.00020	0.00000	0.00040	129269
**SURFACE AREA**											
fusiform	lh	8.311	90937	40414.12	-38.8	-26.6	-23.6	0.00020	0.00000	0.00040	74737
postcentral	lh	4.632	50022	4904.03	-56.4	-19.3	43.7	0.00020	0.00000	0.00040	11644
superiorfrontal	rh	7.596	44472	43511.77	9.0	58.8	26.3	0.00020	0.00000	0.00040	80805

Supplementary Table 4: Structural differences YMs vs. OMs

Significant clusters between young (YMs) and old musicians (OMs) in each of the three cortical measures (thickness, volume, surface area) for both hemispheres respectively. The clusters evolved from the general linear model (GLM) analysis after clusterwise correction for multiple comparisons using Monte Carlo simulations (10000 steps, threshold p=0.05). Annot - annotation of cluster peak, Hemi- hemisphere, lh/rh - left and right hemisphere, Max - indicates the maximum -log10(p-value) in the cluster, VtxMax - vertex number at the maximum, Size - surface area (mm^2) of cluster, MNI X/Y/Z - MNI space (MNI305) coordinate of the maximum, CWP - clusterwise p-value, CWPLow and CWPHi - 90% confidence interval for CWP, NVtxs - number of vertices in cluster

Brain region	Hemi	TAL X	TAL Y	TAL Z	t-peak	t-value	N Voxel
**Contrast YMs > OMs**							
Angular Gyr, BA39	lh	-43	-71	30	6.87	4.38	5173
Inferior Parietal Lobule, BA40	rh	41	-53	45	6.72	4.20	3339
Medial Frontal Gyr, BA6	lh	-1	-8	66	5.92	4.07	618
Cerebellum, Pyramis	lh	-40	-74	-33	6.11	4.03	2593
Cerebellum, Uvula	lh	-7	-80	-36	5.46	4.02	1234
Cerebellum	lh	-25	-53	-27	5.85	4.12	196
Cerebellum, Uvula	rh	5	-83	-33	6.32	4.02	2033
Cerebellum,Culmen	rh	17	-41	-21	5.43	4.00	269
Cerebellum, Inferior Semi	rh	35	-65	-43	4.68	3.79	1008
Cerebellum, Dentate	rh	20	-59	-24	5.71	4.05	236
Middle Frontal Gyr, BA9	rh	50	16	30	5.91	4.15	580
Transverse Temporal Gyr, BA41	rh	50	-17	12	11.01	6.17	4968
Superior Temporal Gyr, BA41	lh	-52	-23	9	15.68	6.22	9400
Insula, BA22	rh	41	-29	0	6.66	5.12	563
Middle Temporal Gyr, BA20	rh	59	-44	-13	6.21	4.44	614
Inferior Temporal Gyr, BA37	lh	-58	-56	-6	4.99	3.82	770
Middle Temporal Gyr, BA21	lh	-68	-17	-12	5.53	4.00	313
Middle Frontal Gyr, BA6	lh	-34	10	51	5.81	3.96	799
Paracentral Lobule, BA5	lh	-4	-29	51	4.40	3.70	244
Cingulate Gyr, BA31	lh	-1	-32	33	4.62	3.73	371
Cingulate Gyr, BA32	lh	-4	19	42	5.60	3.87	960
Superior Frontal Gyr, BA6	rh	2	4	57	4.78	3.75	262
Medial Frontal Gyr, BA6	lh	-10	1	51	5.29	3.92	267
Middle Frontal Gyr, BA6	rh	44	4	45	5.97	3.92	512
Inferior Frontal Gyr, BA*	lh	-52	34	0	4.53	3.76	127
Posterior Cingulate, BA23	lh	-7	-59	18	5.88	3.94	1255
Cingulate Gyr, BA31	rh	14	-53	24	4.48	3.69	220
Middle Frontal Gyr, BA10	lh	-40	43	21	5.43	3.88	254
Precentral Gyr, BA6	lh	-40	-5	39	5.78	3.95	1581
Precentral Gyr, BA4	lh	-58	-2	21	6.45	4.03	221
Posterior Cingulate, BA29	lh	-7	-44	6	5.24	3.99	471
Parahippocampal Gyr, BA30	rh	14	-38	3	4.55	3.71	117
Uncus, BA28	rh	20	4	-27	4.73	3.78	174
Inferior Frontal Gyr, BA47	rh	31	25	-15	4.56	3.83	234
Inferior Frontal Gyr, BA44	lh	-55	16	18	6.09	4.19	947
Precentral Gyr, BA44	lh	-46	10	9	5.27	4.00	563
Superior Frontal Gyr, BA6	lh	-19	22	54	5.37	4.04	432
Postcentral Gyr, BA40	lh	-40	-29	48	6.30	4.41	1436
Inferior Parietal Lobule, BA40	lh	-49	-50	45	5.25	4.20	425
**Contrast OMs > YMs**							
Superior Temporal Gyr, BA22	lh	-49	-20	-3	-9.08	-5.22	684
Postcentral Gyr, BA3	rh	56	-17	24	-5.86	-3.98	302
Cingulate Gyr, BA31	rh	5	-38	42	-4.78	-3.89	144
Claustrum	lh	-28	7	9	-5.64	-3.80	452
Inferior Frontal Gyr, BA44	rh	53	1	18	-5.66	-4.09	131
Inferior Occipital Gyr, BA19	rh	44	-68	-6	-6.17	-4.02	1372
Middle Occipital Gyr, BA37	lh	-49	-71	3	-4.53	-3.76	135
Middle Temporal Gyr, BA37	lh	-52	-62	9	-4.33	-3.70	121
Fusiform Gyr, BA37	lh	-37	-62	-9	-5.51	-4.13	290
Fusiform Gyr, BA37	lh	-40	-41	-15	-4.66	-3.85	139
Fusiform Gyr, BA18	lh	-28	-86	-18	-5.35	-3.84	339
Lingual Gyr, BA18	rh	26	-80	-9	-4.30	-3.66	158
Postcentral Gyr, BA40	lh	-55	-23	21	-7.31	-4.45	142

Supplementary Table 5: YMs vs. OMs functional group contrast

Significant clusters evolved from the functional activation maps of YMs vs. OMs during tone vs. baseline presented with: their anatomical location according to Talairach atlas as well as the corresponding brain region, hemisphere, t-values of the peak voxel, t-value of the clusters mean, number of voxels and Brodmann area, if applicable. The statistical threshold was set at FDR ($p < 0.01$). Hemi - hemisphere, BA - Brodmann area, lh/rh - left and right hemisphere, YMs - young musicians, OMs - old musicians, ONMs - old non-musicians, TAL X/Y/Z - Talairach coordinates, N Voxel - number of voxels in cluster.

Brain region	Hemi	TAL X	TAL Y	TAL Z	t-peak	t-value	N Voxel
**Contrast OMs > ONMs**							
Medial Frontal Gyr, BA6	rh	5	-2	57	5.36	4.16	547
Precentral Gyr, BA6	rh	41	-5	52	7.07	4.65	473
Precentral Gyr, BA6	lh	-25	-11	48	4.97	4.12	214
Middle Frontal Gyr, BA6	rh	29	-8	42	5.19	4.17	121
Inferior Parietal Lobule, BA40	rh	41	-41	45	4.92	4.03	170
Inferior Frontal Gyr, BA46	rh	56	34	6	5.07	4.14	193
Inferior Parietal Lobule, BA40	rh	53	-29	30	5.99	4.49	587
Middle Temporal Gyr, BA37	lh	-46	-53	3	6.20	4.31	678
Inferior Temporal Gyr, BA37	lh	-52	-56	-6	6.38	4.34	297
Supramarginal Gyr, BA40	lh	-37	-41	33	6.25	4.32	446
Inf Semi Lunar Lobule, Cerebellum	lh	-4	-65	-39	5.58	4.23	370
Middle Frontal Gyr, BA46	rh	44	34	15	5.69	4.26	226
Inferior Frontal Gyr, BA44	rh	50	1	15	4.61	4.00	169
Postcentral Gyr, BA3	rh	53	-10	42	6.04	4.45	143
Cingulate Gyr, BA24	lh	-7	1	39	4.93	4.03	172
Precuneus, BA31	lh	-28	-74	18	6.10	4.31	280
Postcentral Gyr, BA40	lh	-55	-23	21	5.45	4.13	157
Medial Frontal Gyr, BA9	rh	26	34	21	4.55	4.01	110
Middle Frontal Gyr, BA10	lh	-37	40	12	6.01	4.26	110
Superior Temporal Gyr, BA41	lh	-43	-26	6	6.13	4.12	302
Lentiform Nucleus, Putamen	lh	-28	7	3	5.32	4.09	1053
Superior Temporal Gyr, BA22	lh	-49	-8	3	7.37	4.75	526
Caudate, Caudate Head	lh	-7	13	3	5.89	4.17	560
Superior Temporal Gyr, BA13	rh	44	-20	9	6.99	4.67	189
Superior Temporal Gyr, BA22	rh	62	-32	9	5.23	4.23	301
Superior Temporal Gyr, BA22	rh	53	-8	6	8.02	4.62	163
Superior Temporal Gyr, BA22	rh	53	13	-3	5.23	4.24	382
Inferior Temporal Gyr, BA20	rh	53	-50	-9	5.52	4.30	567
Middle Occipital Gyr, BA37	rh	47	-65	-6	5.31	4.21	258
Middle Temporal Gyr, BA39	rh	44	-56	6	5.01	4.19	223
Inferior Occipital Gyr, BA18	lh	-34	-89	-15	4.74	3.99	367
Middle Temporal Gyr, BA38	lh	-37	7	-36	5.35	4.21	158
**Contrast ONMs > OMs**							
Superior Temporal Gyr, BA22	rh	44	-38	0	-5.00	-4.13	142
Superior Temporal Gyr, BA41	lh	-40	-35	18	-6.77	-4.63	173
Transverse Temporal Gyr, BA41	rh	35	-35	15	-6.57	-4.55	156
Superior Temporal Gyr, BA41	lh	-52	-23	9	-7.72	-4.83	121

Supplementary Table 6: OMs vs. ONMs functional group contrast

Significant clusters evolved from the functional activation maps of OMs vs. ONMs during tone vs. baseline presented with: their anatomical location according to Talairach atlas as well as the corresponding brain region, hemisphere, t-values of the peak voxel, t-value of the clusters mean, number of voxels and Brodmann area, if applicable. The statistical threshold was set at FDR ($p < 0.01$). Hemi - hemisphere, BA - Brodmann area, lh/rh - left and right hemisphere, YMs - young musicians, OMs - old musicians, ONMs - old non-musicians, TAL X/Y/Z – Talairach coordinates, N Voxel - number of voxels in cluster.

Brain region	Hemi	TAL X	TAL Y	TAL Z	t-peak	t-value	N Voxel
**Contrast YMs > ONMs**							
Inferior Parietal Lobule, BA39	lh	-40	-62	39	5.69	3.91	4681
Inferior Parietal Lobule, BA40	lh	-46	-32	45	6.32	3.98	4195
Inferior Temporal Gyr, BA37	lh	-58	-56	-6	8.42	5.63	1455
Middle Temporal Gyr, BA20	rh	56	-44	-9	6.79	4.24	2101
Inferior Frontal Gyr, BA46	rh	38	34	15	6.53	3.92	2600
Middle Frontal Gyr, BA10	lh	-40	43	15	6.68	4.09	3607
Middle Frontal Gyr, BA9	lh	-40	16	27	5.53	3.94	1208
Inferior Parietal Lobule, BA40	rh	41	-53	45	5.59	3.89	3795
Culmen, Cerebellum	lh	-13	-53	-18	4.56	3.66	654
Cerebellar Lingual, Cerebellum	rh	5	-44	-18	4.38	3.59	109
Cerebellar Tonsil, Cerebellum	rh	5	-44	-36	4.68	3.67	237
Uvula, Cerebellum	rh	11	-80	-36	6.43	3.84	5235
Inferior Semi Lunar Lobule, Cerebellum	lh	-13	-71	-43	6.20	3.92	7655
Middle Temporal Gyr, BA38	lh	-40	7	-39	5.48	3.81	150
Inferior Frontal Gyr, BA46	lh	-49	37	0	4.90	3.68	326
Inferior Frontal Gyr, BA47	lh	-31	28	-6	5.04	3.74	546
Inferior Frontal Gyr, BA45	rh	56	28	3	5.57	3.80	749
Medial Frontal Gyr, BA9	lh	-22	31	27	4.27	3.64	123
Superior Frontal Gyr, BA6	rh	2	-2	66	5.95	4.11	1631
Medial Frontal Gyr, BA6	rh	2	1	54	5.56	3.89	2348
Medial Frontal Gyr, BA8	lh	-1	25	42	5.24	3.83	1899
Superior Frontal Gyr, BA8	rh	14	43	39	4.24	3.56	223
Postcentral Gyr, BA2	lh	-28	-35	60	5.03	3.76	322
Precentral Gyr, BA4	rh	20	-23	57	4.60	3.68	111
Middle Frontal Gyr, BA6	lh	-19	19	54	5.00	3.75	307
Middle Frontal Gyr, BA6	lh	-31	-8	39	6.42	3.89	2844
Precentral Gyr, BA6	rh	44	-5	45	6.85	4.14	2246
Middle Frontal Gyr, BA6	lh	-37	10	48	4.77	3.71	1182
Superior Frontal Gyr, BA8	rh	14	28	48	4.66	3.66	192
Precuneus, BA19	rh	32	-68	36	5.19	3.84	842
Precentral Gyr, BA4	lh	-58	-5	21	6.40	4.00	977
Lentiform Nucleus, Putamen	lh	-16	-2	12	6.07	3.97	718
Lentiform Nucleus, Medial Globus Pallidus	lh	-10	-2	0	4.93	3.71	309
Caudate, Caudate Body	rh	11	-2	15	5.23	3.73	1195
Cingulate Gyr, BA31	lh	-4	-32	30	6.32	4.07	3630
Medial Frontal Gyr, BA6	lh	-1	-23	57	4.93	3.73	410
Posterior Cingulate, BA29	lh	-7	-47	9	5.76	3.78	1940
Precuneus, BA31	lh	-4	-68	21	4.57	3.70	701
Insula, BA13	lh	-43	7	12	4.65	3.58	296
Superior Temporal Gyr, BA22	lh	-56	13	0	4.64	3.72	124
Inferior Frontal Gyr, BA44	lh	-61	17	15	4.19	3.50	123
Superior Frontal Gyr, BA10	lh	-7	61	18	4.21	3.58	430
Inferior Parietal Lobule, BA40	lh	-52	-41	27	5.05	3.78	498
Caudate, Caudate Tail	rh	29	-32	12	6.43	4.14	165
Precentral Gyr, BA44	rh	50	10	12	7.07	4.52	2022
Superior Temporal Gyr, BA41	rh	44	-41	12	7.77	5.18	1074
Superior Temporal Gyr, BA41	lh	-46	-23	6	14.27	7.04	7951
Claustrum	lh	-37	-17	-3	6.86	4.33	851
Superior Temporal Gyr, BA13	rh	44	-20	9	15.19	5.42	13452
**Contrast ONMs > YMs**							
Superior Temporal Gyr, BA22	lh	-49	-20	-3	-8.01	-4.63	854
Lingual Gyr, BA18	rh	35	-68	-6	-5.51	-3.88	1204
Temporal Subgyral, BA21	rh	44	-11	-9	-5.57	-3.83	156

Supplementary Table 7: YMs vs. ONMs functional group contrast

Significant clusters evolved from the functional activation maps of YMs vs. ONMs during tone vs. baseline presented with: their anatomical location according to Talairach atlas as well as the corresponding brain region, hemisphere, t-values of the peak voxel, t-value of the clusters mean, number of voxels and Brodmann area, if applicable. The statistical threshold was set at FDR ($p<0.01$). Hemi - hemisphere, BA - Brodmann area, lh/rh - left and right hemisphere, YMs - young musicians, OMs - old musicians, ONMs - old non-musicians, TAL X/Y/Z – Talairach coordinates, N Voxel - number of voxels in cluster.

ROI	Hemi	Meas	Group					
			**YMs**		**OMs**		**ONMs**	
			mean	SD	mean	SD	mean	SD
**STG**	**lh**	**THK**	2.97	0.13	2.54	0.14	2.67	0.13
**SMG**	**rh**	**VOL**	11458.13	1432.48	8920.75	1353.34	9194.93	1361.11
**aHG**	**rh**	**THK**	2.46	0.19	2.02	0.32	2.18	0.25
**Cuneus**	**rh**	**SA**	1613.31	175.29	1436.75	101.36	1325.93	182.56

Supplementary Table 8: Preselected structural ROIs
Average group values of thickness, volume, surface area in preselected structural ROIs resulted significant in the discriminant function analysis. The structural ROIs are part of the FreeSurfer Desikan atlas. ROI - region of interest, Hemi - hemisphere, lh/rh - left and right hemisphere, Meas - Measures, YMs - young musicians, OMs - old musicians, ONMs - old non-musicians, SD - standard deviation, THK - thickness, VOL - volume, SA - surface area, STG - superior temporal gyrus, SMG - supramarginal gyrus, aHG - anterior Heschl's Gyrus.

Analysis Level 2: Differential	Predicting Brain Area (descending relevance)	Hemi	Meas	Wilk's	CCA	df	p	Hit rate	r
	**Destrieux atlas**								
**OMs vs. ONMs**	Precuneus	rh	VOL	0.47	0.73	7	0.01	93.5	0.58
	Temporal Pole	lh	THK						0.49
	Posterior-dorsal cingulate gyrus (dPCC)	lh	THK						0.39
	Sulcus of Jensen	rh	THK						0.34
	Planum temporale	lh	SA						0.33
	Planum polare	rh	THK						0.25
	Supramarginal gyrus	lh	THK						0.14
**YMs vs. ONMs**	Posterior-dorsal cingulate gyrus (dPCC)	lh	THK	0.18	0.91	7	0.00	96.8	0.52
	Precuneus	rh	VOL						0.49
	Planum polare	rh	THK						0.44
	Supramarginal gyrus	lh	THK						0.35
	Planum temporale	lh	SA						0.25
	Sulcus of Jensen	rh	THK						0.25
	Temporal Pole	lh	THK						0.11
**YMs vs. OMs**	Precuneus	rh	VOL	0.15	0.92	7	0.00	100	0.68
	Posterior-dorsal cingulate gyrus (dPCC)	lh	THK						0.59
	Sulcus of Jensen	rh	THK						0.49
	Planum polare	rh	THK						0.4
	Supramarginal gyrus	lh	THK						0.4
	Temporal Pole	lh	THK						0.3
	Planum temporale	lh	SA						0.13

Supplementary Table 9: Significant structural ROIs from discriminant function analysis

Results of the two-group level discriminant function analysis of the structural ROIs. The specific ROIs are from FreeSurfer Destrieux atlas. These clusters are also depicted in **Supp.Fig.2**. Hemi - hemisphere, lh/rh - left and right hemisphere, YMs - young musicians, OMs - old musicians, ONMs - old non-musicians, THK - thickness, VOL - volume, SA - surface area, ROI - region of interest, r - Contribution to discriminant function, hit rate - discrimination accuracy, Meas - measure, Wilks’ - Wilks Lamda test, CCA - canonical correlation analysis, df - degrees of freedom.

**Measure**	**Test**	**Reference**	**Duration (min)**
Cognitive status	MOCA	Nasreddine et al. (2019)	10
Executive function, task-switching	Trail-making-test A&B	Reitan and Wolfson (1985)	5
Processing speed	Simple Reaction Time Task (PC-Test)	Ghisletta et al. (2018)	5
Verbal working memory (WM) - Updating	Verbal 2-back task (PC-Test)	Test battery from Memory clinic, Department of Geriatric Medicine, Felix Platter (FPS), Basel, Switzerland	10
Verbal Short-Term-Memory (STM) / WM	Verbal span (forward/ backward)	From WMS-R: Klaiberg (2003)	5
Visual STM / WM	Visual span (forward/ backward)	From WMS-R: Klaiberg (2003)	5
Interference susceptibility	Stroop Test	Tremblay et al. (2016)	5
Fluid intelligence	Raven's standard progressive matrices	Burke (1958)	20
Single and dual gait and cognition - tasks	GAITRite© electronic walkway system / simple gait	Test battery from Basel Mobility Center (BMC), Felix Platter (FPS), Basel, Switzerland	20-30
Distant visual acuity	E-table	Test battery from Basel Mobility Center (BMC), Felix Platter (FPS), Basel, Switzerland	3
Near visual acuity	Landolt C / Landoltrings	Test battery from Basel Mobility Center (BMC), Felix Platter (FPS), Basel, Switzerland	3
Hand strength	Martin Vigorimeter	Sipers et al. (2016)	2
Musicality	AMMA	Gordon et al. (2018)	12
Audiogram	AFC	http://medi.uni-oldenburg.de/afc	5-10
Cognitive reserve	Questionnaire	Forstmeier and Maercker (2009); Sattler et al. (2012)	5-10
Depression	Geriatric Depression Scale	Gauggel and Birkner (1999)	5-10
Handedness	Handedness questionnaire	Annett (1967)	5-10
Subjective wellbeing health	Questionnaire (SF-36 Version1)	Test battery from Basel Mobility Center (BMC), Felix Platter (FPS), Basel, Switzerland	5-10

Supplementary Table 10: Overview of all performed tests to assess cognitive, mental and medical health of participants

Frequency (kHz)	OMs mean	OMs SEM	ONMs mean	ONMs SEM	t	df	p
**0.4**	-22.17	6.56	-20.37	5.12	0.22	28	0.830
**0.7**	-9.25	2.65	-14.63	3.55	-1.22	28	0.234
**1.0**	-10.48	3.02	-12.10	3.88	-0.33	28	0.745
**2.0**	-22.05	3.74	-15.22	3.58	1.32	28	0.197
**3.0**	-30.72	4.42	-29.42	4.45	0.21	28	0.837
**4.0**	-45.43	4.59	-39.68	4.55	0.89	28	0.381
**5.0**	-52.63	3.75	-45.92	4.77	1.11	28	0.277
**6.0**	-56.15	4.50	-48.85	3.68	1.25	28	0.220

OMs – old musicians, ONMs – old non-musicians

Supplementary Table 11: Statistical results of hearing assessment in elderly participants

References

Annett, M. (1967). The binomial distribution of right, mixed and left handedness. *Q J Exp Psychol* 19(4)**,** 327-333. doi: 10.1080/14640746708400109.

Benner, J., Wengenroth, M., Reinhardt, J., Stippich, C., Schneider, P., and Blatow, M. (2017). Prevalence and function of Heschl's gyrus morphotypes in musicians. *Brain Struct Funct* 222(8)**,** 3587-3603. doi: 10.1007/s00429-017-1419-x.

Burke, H.R. (1958). Raven's progressive matrices: a review and critical evaluation. *J Genet Psychol* 93(2)**,** 199-228. doi: 10.1080/00221325.1958.10532420.

Forstmeier, S., and Maercker, A. (2009). Potentially modifiable risk factors in the development of Alzheimer’s disease. *European Neurological Review* 4(1)**,** 18-21.

Gauggel, S., and Birkner, B. (1999). Validity and reliability of a German version of the Geriatric Depression Scale (GDS). *Zeitschrift Fur Klinische Psychologie-Forschung Und Praxis* 28(1)**,** 18-27. doi: Doi 10.1026//0084-5345.28.1.18.

Ghisletta, P., Renaud, O., Fagot, D., Lecerf, T., and de Ribaupierre, A. (2018). Age and sex differences in intra-individual variability in a simple reaction time task. *International Journal of Behavioral Development* 42(2)**,** 294-299. doi: 10.1177/0165025417739179.

Gordon, C.L., Cobb, P.R., and Balasubramaniam, R. (2018). Recruitment of the motor system during music listening: An ALE meta-analysis of fMRI data. *PLoS One* 13(11)**,** e0207213. doi: 10.1371/journal.pone.0207213.

Klaiberg, A. (2003). Wechsler Gedachtnistest-Revidierte Fassung von C. Härting, HJ Markowitsch, H. Neufeld, P. Calabrese, K. Deisinger & J. Kessler (2000). *Diagnostica* 49(1)**,** 45-47. doi: 10.1026//0012-1924.49.1.45.

Nasreddine, Z.S., Phillips, N.A., Bedirian, V., Charbonneau, S., Whitehead, V., Collin, I., et al. (2019). The Montreal Cognitive Assessment, MoCA: A Brief Screening Tool For Mild Cognitive Impairment (vol 53, pg 695, 2005). *Journal of the American Geriatrics Society* 67(9)**,** 1991-1991. doi: 10.1111/jgs.15925.

Reitan, R.M., and Wolfson, D. (1985). *The Halstead-Reitan neuropsychological test battery: Theory and clinical interpretation.* Reitan Neuropsychology.

Sattler, C., Toro, P., Schonknecht, P., and Schroder, J. (2012). Cognitive activity, education and socioeconomic status as preventive factors for mild cognitive impairment and Alzheimer's disease. *Psychiatry Res* 196(1)**,** 90-95. doi: 10.1016/j.psychres.2011.11.012.

Sipers, W.M., Verdijk, L.B., Sipers, S.J., Schols, J.M., and van Loon, L.J. (2016). The Martin Vigorimeter Represents a Reliable and More Practical Tool Than the Jamar Dynamometer to Assess Handgrip Strength in the Geriatric Patient. *J Am Med Dir Assoc* 17(5)**,** 466 e461-467. doi: 10.1016/j.jamda.2016.02.026.

Tremblay, M.P., Potvin, O., Belleville, S., Bier, N., Gagnon, L., Blanchet, S., et al. (2016). The Victoria Stroop Test: Normative Data in Quebec-French Adults and Elderly. *Arch Clin Neuropsychol* 31(8)**,** 926-933. doi: 10.1093/arclin/acw029.
